# LINEs of evidence: noncanonical DNA replication as an epigenetic determinant

**DOI:** 10.1186/1745-6150-8-22

**Published:** 2013-09-13

**Authors:** Ekaterina Belan

**Affiliations:** 1 Genetics Laboratory, Royal University Hospital, Saskatoon, SK S7N 0W8, Canada

**Keywords:** LINE-1, L1 retrotransposon, DNA replication, Replication timing, Epigenetics, Pluripotency, Cancer, Embryonic stem cells, Chromatin domains, Origins of replication

## Abstract

LINE-1 (L1) retrotransposons are repetitive elements in mammalian genomes. They are capable of synthesizing DNA on their own RNA templates by harnessing reverse transcriptase (RT) that they encode. Abundantly expressed full-length L1s and their RT are found to globally influence gene expression profiles, differentiation state, and proliferation capacity of early embryos and many types of cancer, albeit by yet unknown mechanisms. They are essential for the progression of early development and the establishment of a cancer-related undifferentiated state. This raises important questions regarding the functional significance of L1 RT in these cell systems. Massive nuclear L1-linked reverse transcription has been shown to occur in mouse zygotes and two-cell embryos, and this phenomenon is purported to be DNA replication independent. This review argues against this claim with the goal of understanding the nature of this phenomenon and the role of L1 RT in early embryos and cancers. Available L1 data are revisited and integrated with relevant findings accumulated in the fields of replication timing, chromatin organization, and epigenetics, bringing together evidence that strongly supports two new concepts. First, noncanonical replication of a portion of genomic full-length L1s by means of L1 RNP-driven reverse transcription is proposed to co-exist with DNA polymerase-dependent replication of the rest of the genome during the same round of DNA replication in embryonic and cancer cell systems. Second, the role of this mechanism is thought to be epigenetic; it might promote transcriptional competence of neighboring genes linked to undifferentiated states through the prevention of tethering of involved L1s to the nuclear periphery. From the standpoint of these concepts, several hitherto inexplicable phenomena can be explained. Testing methods for the model are proposed.

**Reviewers:**

This article was reviewed by Dr. Philip Zegerman (nominated by Dr. Orly Alter), Dr. I. King Jordan, and Dr. Panayiotis (Takis) Benos. For the complete reviews, see the Reviewers’ Reports section.

## Review

### Introduction

L1 elements have propagated in mammalian genomes by means of autonomous retrotransposition. Retrotransposition of an L1 element occurs through reverse transcription of its RNA intermediate and subsequent insertion of an L1 cDNA copy at a new location in the genome [1]. As a result of such propagation, L1s comprise ~17%, ~19%, and ~23% of the human, mouse, and rat genome, respectively [2-4]. Among the 516,000 L1 sequences identified in the draft human genome, the majority of the elements are truncated (usually at the 5′ end) L1 copies [2]. Only 7046 L1 sequences in the reference human genome are full-length L1 (FL-L1) elements [5], 1000 of which have been classified as potentially active [2] in terms of retrotransposition. Although only ~80–100 active FL-L1s belonging to the L1Hs subfamily are thought to be present in the reference human genome [6], active FL-L1s seem to be more abundant in individual genomes [7]. Human FL-L1s are similar in length (~6 kb) but heterogenous in sequence composition [5]. This heterogeneity results in a spectrum of functional capabilities of FL-L1s, ranging from the inability to translate the encoded proteins to highly active forms in terms of retrotransposition [8]. However, it remains unexplored whether any retrotransposition inactive FL-L1s are capable of reverse transcription *in vivo*.

A human FL-L1 element contains a 5′ untranslated region (UTR), two open reading frames (ORF1 and ORF2), and a 3′ UTR followed by an A-rich tail [9]. The L1 5′ UTR houses the sense (the first 100 bp) [10] and antisense (positions 400–600) [11] promoters. Transcription from the antisense promoter is one of the known mechanisms involved in L1 silencing, and is thought to promote the downregulation of transcription from the L1 sense promoter because the resultant bidirectional transcripts are processed into small interfering RNAs (siRNAs) [12]. L1s with intact ORFs encode two proteins: ORF1p, a nucleic acid chaperone, and ORF2p, which possesses endonuclease (EN) and RT activities [reviewed in [8]]. Both proteins tend to associate with their encoding RNA [13], forming an L1 ribonucleoprotein (RNP) complex that acts as a molecular machinery of retrotransposition [reviewed in [1]].

It has long been thought that a substantially increased retrotransposition rate is linked to a noticeable synthesis of FL-L1 transcripts and, therefore, occurs in preimplantation embryos [14], several transformed cell lines [15-17], and early meiotic spermatocytes [18]. However, recent evidence shows that retrotransposition occurs mainly in early embryonic and cancerous cells, not in the germline [19-21]. This suggests that the production of FL-L1 RNA *per se* is not sufficient for retrotransposition, and the factors that allow for retrotransposition in embryos but not in the germ cell line remain unknown.

Since the acknowledgement of Barbara McClintock’s discovery of mobile genetic elements [22], the transposition and retrotransposition of these elements have been a major research focus in this field. L1s have successfully propagated in the course of co-evolution with their hosts’ genomes, whereas diverse mechanisms have evolved at the genome level to repress the activity of L1s [[8] and references therein]. Given that L1s constitute one fifth of the genome, it is logical to surmise that their co-evolution with the hosts’ genomes has led not only to the evolvement of an effective defence system against retrotransposition but also to harnessing of L1s for genome functioning. In this regard, the mechanisms by which L1s contribute to genome functioning remain largely unexplored. It is also not known whether the ongoing insertional mutagenesis is linked to some programmed L1-dependent processes in the nucleus.

Some efforts have been made to understand the biological significance of the abundance of L1s in the genome in the context of functionally meaningful elements and the abundance of L1 transcripts in particular cell types. LINEs constitute a substantial portion of scaffold/matrix attachment regions (S/MARs) in the human genome [23]. S/MARs play an essential role in the organization of chromatin as functional loop domains and thus in the regulation of transcription and DNA replication [24,25]. This suggests that numerous L1s may regulate transcription and DNA replication through their involvement in the establishment of the three-dimensional (3D) structure of chromatin. On the other hand, abundantly expressed FL-L1s are known to globally influence gene expression profiles, differentiation state, and proliferation capacity of early embryos and many types of cancer, although by mechanisms which remain unclear [26]. Thus far, the S/MAR-related function of L1s remains unexplored in conjunction with their expression status. The global nature of cellular processes controlled by abundantly expressed FL-L1s suggests that an integrative approach is required to study the functional role of upregulated FL-L1s. Specifically, the role of upregulated FL-L1s should be investigated in a broad context of spatio-temporal organization and functioning of the genome and chromatin.

An important point in this regard is that the involvement of FL-L1 transcripts in the global regulation of early development and carcinogenesis seems to be mediated by L1 RT [26]. This raises the question as to whether substantial L1-related reverse transcription exists in early embryonic and cancer cell systems and, if so, what role it plays. A massive nuclear L1-linked reverse transcription of unknown functional significance has been reported in the mouse zygote and two-cell embryo, which is believed to be DNA replication independent [27]. However, this review will argue that the available data do not allow for definite conclusions regarding whether or not this L1-linked DNA synthesis by reverse transcription is part of the genomic DNA replication/duplication program. Therefore, it is very important to address this question experimentally.

In this review, an attempt is made to fathom how upregulated FL-L1s and their RT globally influence the differentiation state and proliferation capacity of early embryos and many types of cancer. In this context, the most intriguing phenomenon to be explored is the massive nuclear L1-linked reverse transcription found at the onset of embryogenesis. It is difficult, if not impossible, to explain the global epigenetic role of L1 RT and the nature of massive L1-linked reverse transcription within the framework of current concepts. Therefore, conceptual advance is the main challenge. Herein, available L1 data are revisited and examined in concert with relevant findings from the fields of replication timing, chromatin organization, DNA topology, and epigenetics. The broad picture that emerges from this integrative approach favors two novel fundamental concepts. First, noncanonical replication of a portion of genomic FL-L1s by means of L1 RNP-driven reverse transcription is likely to co-exist with DNA polymerase-dependent origin-based replication of the rest of the genome during the same round of DNA replication in embryonic and cancer cell systems. Second, the role of this mechanism is likely epigenetic. Moreover, endogenous retrotransposition may be associated, to a great extent, with failure of this noncanonical DNA replication of an L1 unit. An exploration of this hypothesis shows that the mechanism of DNA replication is worthy of being retested for specific genomic locations (distinct FL-L1 sequences) in mammalian early embryonic and cancer cell systems. This is important to advance understanding of DNA replication, the biology of L1s, and mechanisms of pluripotency and carcinogenesis.

### L1 RNA and RT are essential for early embryogenesis and carcinogenesis

L1 RNAs and RT, abundantly expressed in preimplantation embryos and some cancer cell lines, have been targeted in numerous experiments to investigate their potential roles. These experiments have brought about very important but overlooked findings. Specifically, they demonstrate that the functional knockdown of L1 expression via L1-specific RNA interference (RNAi) and the inhibition of RT both independently result in the same biological outcomes [26,28]. This suggests that both transcription and reverse transcription of L1s are links in the same chain in these cell systems.

The expression of L1s has been shown to be involved in the establishment of an undifferentiated state and a high proliferation rate upon malignant transformation of cells. For example, the knockdown of L1 expression by L1 ORF2-specific antisense oligonucleotides drastically inhibited ^3^H-thymidine incorporation in a dose-dependent manner in human transformed hepatoma (Hep3B) cells [28]. In the human A-375 melanoma cell line, both transient and stable silencing of L1s by ORF1-specific RNAi caused a 50–70% decrease in proliferation rate and promoted differentiation, as was evident from morphological changes and the expression of specific markers [29,30]. The transcription of the proliferation markers *CCND1* and *MYC* was downregulated in A-375 derivative cells upon L1 silencing [30]. Moreover, both transient and stable downregulation of L1 expression in A-375 cells strongly reduced their tumorigenicity when the cells were inoculated in athymic nude mice [26,30]. Notably, the targeting of L1 ORF1 by RNAi in melanoma cells was concomitant with the drastic reduction of translated ORF2p and RT activity in these cells [29,30]. Therefore, it is logical to assume that the observed phenomena are linked to the transcription and subsequent translation of FL-L1s.

The studies performed in early mouse embryos have shown that L1 transcripts are indispensable for the onset of embryogenesis [31]. When antisense oligonucleotides targeting the 5′ UTR and ORF1 of the T_F_ subfamily of FL-L1s were microinjected into the male pronucleus 18–20 h after fertilization, a complete and irreversible arrest of development occurred at the two- or, to a lesser extent, four-cell stage [31]. Despite the arrested development, the microinjected embryos remained viable and morphologically normal for several days. However, microinjection of an ORF2-specific oligonucleotide neither arrested embryonic development nor decreased the RT activity, probably due to a depletion of injected oligonucleotides through the targeting of 5′-truncated L1 transcripts [31]. In contrast, continuous exposure of Hep3B cells to the oligonucleotide present in the culture media [28] could be an effective means to target L1 RNA by ORF2-specific RNAi. Despite the ineffectiveness of the ORF2-specific oligonucleotide at arresting development, the fact that the effect caused by the other two types of oligonucleotides coincided with a significant decrease of the endogenous RT activity [31] suggests that FL-L1 transcripts are essential for the onset of embryogenesis.

An important question is to whether the role of FL-L1s in early embryos and transformed cell lines is due to their transcription *per se* or also due to the involvement of L1-encoded RT. However, the lack of an L1 RT-specific inhibitor, the questionable effectiveness of available anti-RT drugs, and the abundance of RT expressed from endogenous retroviruses (ERVs) in embryonic and cancer cells [32-34] make this task methodologically challenging. For this reason, the effects of downregulated expression of L1s versus ERVs have been compared [26].

Nevirapine, a non-nucleoside RT inhibitor that inhibits endogenous RT, affects early embryos and cancer cell lines in a manner similar to the L1-specific RNAi [26,29,35-37]. The exposure of mouse late zygotes and two- and four-cell stage embryos to nevirapine caused developmental arrest at the preimplantation stages [35]. The effect of nevirapine was dose-dependent, and the arrested blastomeres maintained normal morphology after several days in culture [35]. However, nevirapine did not cause developmental arrest being added to early zygotes (the first 5 hr after fertilization) and later embryos (from the eight-cell stage onwards) [35]. Exposure of a variety of human and murine tumor cell lines to nevirapine quickly reprogrammed them to differentiating derivatives: the cells exhibited drastically decreased proliferation rates, globally changed expression profiles of several hundred genes, and downregulated expression of *CCND1* and *MYC* [[26] with a reference to unpublished data, [29,30,38]]. Additionally, nevirapine induced the expression of cell-type-specific differentiation markers in many transformed cell lines, including the genetically abnormal acute myeloid leukemia (AML) cell lines with t(15;17) *PML*/*RARA* and t(8;21) *AML1*/*ETO* and primary blasts from AML patients [29,37,38]. Interestingly, the effect of nevirapine was irreversible in early embryos [36] but reversible in tumor cells [29,37,38].

The inhibition of telomerase RT is reasoned to be an unlikely cause of these phenomena [29,35]. However, the interpretation of the nevirapine-caused effects as L1 RT-dependent was questioned because nevirapine was an ineffective inhibitor of L1 RT in cell-based retrotransposition assays [39,40]. Nevirapine was ineffective when tested on an FL-L1 element [39] at much lower concentrations than were effective in the reprogramming of transformed cells [38]. The ineffectiveness of nevirapine at inhibiting the synthetic L1 RT [40] could be attributed to conformational changes of the inhibitor binding “pocket”, which could arise in this protein made of the L1 RT domain and a non-L1 segment and post-translationally modified in non-mammalian cells. Despite being ineffective at inhibiting retrotransposition in these assays, nevirapine nevertheless completely blocked RT activity when tested on lysates of F9 mouse teratocarcinoma cells [38], which are known to actively express FL-L1s [16]. Efavirenz, another non-nucleoside RT inhibitor, decreased the proliferation rate and promoted the differentiation of cancer cell lines in a manner akin to nevirapine [29,37]. It also was found to be an effective L1 RT inhibitor in *in vitro* retrotransposition assays when used at similar concentrations [40]. Taken together, these data suggest that although nevirapine seems to be a less potent L1 RT inhibitor than efavirenz, it can inhibit endogenous L1 RT when used at high concentrations.

If nevirapine does inhibit L1 RT *in vivo*, the unresponsiveness of early zygotes, known to have L1 RT carried over by the spermatozoid [27], and late pre-implantation embryos, which also actively express FL-L1s [14], requires further investigation. It can be hypothesized that the presence of a noticeable lag period between the onset of the exposure of two- and four-cell embryos to nevirapine and the developmental arrest [35] is because the presynthesized L1 RT was incapable of binding this drug. The unresponsiveness of blastocysts to nevirapine could also be because the concentration of nevirapine reaching cells of the inner cell mass (ICM) was too low to cause noticeable effects.

Actively transcribed and reverse transcribed L1s, rather than ERVs, are thought to be a driving force of tumorigenic reprogramming and early development progression [26]. L1-interfered A-375 cells exhibited a downregulated expression of HERV-K, the biologically most active family of human ERVs, whereas a functional knockdown of HERV-K did not affect the level of L1 expression, proliferation rate, or phenotype [30]. Consistent with this observation, downregulated expression of murine endogenous retrovirus-like element (MuERV-L) in mouse zygotes caused only mild and transient suppression of development [41]. Similarly, stable knockdown of expression of ERVs in early cloned transgenic pig embryos did not interfere with normal embryonic and post-natal development [42].

The phenomenon of massive nuclear reverse transcription coinciding with a two-fold increase of L1 DNA copy number in the mouse zygote and the two-cell embryo as well as the transient nature of this increase (it diminishes in blastocysts) [27], strongly suggests that L1 RNA is actively and transiently reverse transcribed in preimplantation embryos. This nuclear reverse transcription could be due to L1 RT rather than ERV RT because, based on current knowledge, ERVs are reverse transcribed in the cytoplasm [43].

Attempts to explain how L1 transcription and reverse transcription can be implicated in fundamental biological processes in early embryos and cancers have not yet brought about any concrete and plausible model. Dr. Spadafora and colleagues hypothesize that both L1-dependent transcriptional interference and non-random retrotransposition events that are followed, at least in embryos, by the excision of a portion of newly inserted L1 copies might have a role in these cell systems [27,36,38]. However, the term “transcriptional interference”, defined as the activity of one transcriptional unit modified by the activity of another [44], does not specify the molecular mechanism. Furthermore, the fact that the addition of anti-RT drugs to cancer cell lines quickly reprograms them to “normal” phenotypes, and their withdrawal abolishes this effect, does not favor the hypothesis of genetic changes. It is unlikely that L1 RT regulates fundamental cellular processes by massive retrotransposition in embryos and by another means in cancers. Spadafora [36] also hypothesized that L1 RT could be implicated in the substantial repositioning of chromatin in the nuclei and, therefore, in the modulation of expression of other genes. This assumption was made based on unpublished data, obtained in his laboratory, that suggest the nuclei of nevirapine-exposed F9 teratocarcinoma cells undergo a reorganization of their functional compartments. However, no molecular mechanism has been proposed to explain how L1 RT can be involved in chromatin reorganization.

A model to explain how L1 RNAs and RT are implicated in the fundamental processes in early embryos and certain cancers must address several issues. Specifically it should: (i) demonstrate the utility of expressed L1 RNAs, ORF1p, and ORF2p, taking into consideration that ORF2p acts as an RT, synthesizing cDNA; (ii) explain why early embryos stop dividing, but transformed cells do not show a complete lack of proliferation in response to L1-specific RNAi or RT inhibition; (iii) explain why the inhibition of RT (most likely L1 RT as discussed above) causes irreversible arrest of early embryonic development versus the reversible effects in cancer cell lines; and (iv) describe how downregulation of L1s can reprogram dedifferentiated cancer cells to their original cell types but not to other cell types. An attempt to address these issues is made below.

### L1 elements and replication timing programs in pluripotent and cancerous cells

The results of the insightful studies by Dr. Gilbert’s laboratory on changes in replication timing and chromatin organization linked to the loss of pluripotency in differentiating embryonic stem cells (ESCs) [45,46] might shed light on the role of upregulated L1s in establishing an undifferentiated state in a cell.

The replication-timing program is the order in which different chromosomal domains are replicated during S phase [47]. Genome-wide profiling of replication timing in numerous cell types in mouse and human have indicated that chromosomes consist of alternating early and late S replicating domains [45,48-50]. Multi-megabase replication domains are prevalent in differentiated cells, whereas alternating small (400–800 kb) early and late S replication domains are well represented in mouse and human pluripotent ESCs and in mouse induced pluripotent stem cells (iPSCs) [45,49]. Importantly, the replication timing profile of the genome is a dynamic, developmentally regulated feature that is coordinated with the reprogramming of gene expression and repositioning of chromosome domains within the nucleus [45,46,51]. Differentiation of mouse pluripotent ESCs to neural precursor cells (NPCs) is associated with replication timing changes that affect approximately 20% of the genome [45].

There has been an attempt to determine whether pluripotency is associated with distinct features of a replication timing profile in a genomic context [45]. Two features of a replication timing profile were originally considered to be characteristic of pluripotent cells [45]. One was the presence of small domains that change replication timing from early in ESCs to late in NPCs (EtoL) and, vice versa, from late to early (LtoE). These changes result in the merging of small domains into larger, coordinately replicating domains with a consequent 40% reduction in number. The interruption of late replicating L1-rich AT isohores by small early replicating (EtoL) domains and early replicating L1-poor GC isohores by late replicating (LtoE) domains was also thought to be a feature associated with pluripotency [45]. However, it has become evident that the consolidation of replication domains and their alignment to AT and GC isochores were more specific to the formation of ectoderm than mesoderm and endoderm [46]. Moreover, the improvement of the correlation of replication timing to GC/L1 content was weaker in differentiating human versus mouse ESCs [49]. In terms of replication timing features, the most notable “fingerprint” or “indicator” of pluripotency in mice was found to be the presence of early S replicating domains that reside in a subset of L1-rich (~27.5%)/AT-rich (~59.7%) isochores with an unusually high (for AT isochores) density of genes [45,51]. The large EtoL replication-timing switches of these domains are strongly associated with loss of pluripotency [45,51].

A study of replication timing and transcription profiles of a variety of independent cell lines representing different stages of early mouse embryogenesis [46] has revealed that (i) loss of pluripotency is associated with a number of EtoL replication-timing changes, which are lineage-independent and completed by the late post-implantation epiblast stage prior to germ layer specification and are stably maintained in all downstream lineages; (ii) these EtoL changes precede the downregulation of key pluripotency transcription factors [POU5F1 (also known as OCT4)/NANOG/SOX2]; (iii) these EtoL replication-timing changes tend to be accompanied by a repositioning of these domains toward the nuclear periphery and a downregulation of genes residing in these segments, especially those with low CpG density promoters; (iv) the completion of lineage-independent EtoL changes coincides with a transition of these EtoL domains to a stable silent epigenetic state, which is very difficult to reprogram back to the pluripotent state in terms of replication timing and the expression of genes with low CpG density promoters; (v) DNA methylation of genes with low CpG density promoters within these EtoL domains and activity of several chromatin modifying enzymes are not a main cause of the established irreversibility; (vi) the acquired stable silencing of lineage-independent EtoL domains on autosomes is reminiscent of the irreversible heterochromatinization of the inactive X chromosome (Xi) in female mammals and occurs within the same time frame in development; (vii) the subnuclear repositioning of EtoL domains occurs in parallel with a dramatic switch to chromatin compaction along the nuclear envelope; and (viii) these lineage-independent EtoL domains represent 6.1% or 155 Mb of the genome. Interestingly, lineage-dependent EtoL and LtoE changes, occurring after the late epiblast stage, are easier to reprogram back than lineage-independent EtoL switches. An important conclusion from this study is that loss of pluripotency is associated with establishing a very stable epigenetic barrier in the absence of large-scale transcription changes, and that these epigenetic changes are mapped to lineage-independent L1-rich/gene-rich EtoL domains [46].

It is largely unknown what mechanism drives replication timing changes during loss of pluripotency and exactly what forces the pluripotency “indicator” domains to replicate early in ESCs. Rif1 protein has been recently identified as a key determinant that establishes the replication timing program and the size of replication domains in mouse embryonic fibroblasts and in human transformed HeLa cells [52,53]. Rif1 is thought to perform this role by attaching certain chromatin segments to the nuclear matrix and establishing restricted access to the Rif1-bound segments for replication factors in early S phase [52,53]. *Rif1* expression is developmentally regulated [54]; however, the functional significance of the expression patterns and a correlation with pluripotency are not understood. Although Rif1 is highly expressed in totipotent and many pluripotent cell types (zygotes, cleaving embryos, ESC lines maintained *in vitro*, primordial germ cells), it is downregulated in the ICM of the blastocyst [54]. Rif1 becomes downregulated by the downregulation of OCT4 and NANOG [55]. Knockdown of *Rif1* leads to differentiation of ESCs [55], which suggests that Rif1 is implicated, at least to some extent, in the maintenance of a pluripotency-specific replication timing profile. However, the lack of a strong correlation between *Rif1* expression and pluripotency [54], the fact that Rif1 mainly regulates mid-S replication domains, and its role as a preventer and not a promoter of early-S replication [52,53] suggest that this protein is unlikely to provide early-S replication of the EtoL pluripotency “indicator” domains.

A number of observations suggest that late replication is the default state of EtoL developmentally regulated domains, and that an additional as yet unknown property must be imposed upon these domains in order to switch them to the early replication state [50]. It is worth mentioning that no one has sought to discover whether the active transcription of L1s, found in both human and mouse ESCs [45,56,57], plays a role in the early replication of L1-rich EtoL domains. In this regard, I propose that specific subsets of FL-L1 transcripts, if present, allow for the early replication and euchromatinization of the EtoL domains to which they map. The downregulation of this transcription may trigger EtoL replication timing switches and cause the heterochromatinization of the corresponding domains, thus contributing to loss of pluripotency. The downregulation of transcription of a different subset of L1s might be involved in loss of totipotency. This idea is supported by the fact that loss of either totipotency or pluripotency coincides with a wave of chromatin compaction near the nuclear periphery in the absence of large-scale changes of transcription profiles [46,58]. Uniformly dispersed chromatin fibers of the pronuclei undergo dramatic reorganization in two- and four-cell stage embryos when heterochromatin blocks emerge near the nuclear envelope, nucleolar precursor body, and in the nuclear interior [58,59]. The first wave of heterochromatinization is associated with the loss of totipotency that occurs by the eight-cell stage [60]. It is followed by a conversion of chromatin to a highly dispersed conformation in pluripotent cells but not in the lineage-restricted trophectoderm and primitive endoderm of the blastocyst [58]. The second wave of chromatin compaction near the nuclear periphery is linked to the loss of pluripotency [46]. It is tempting to speculate that, in both cases, similar epigenetic barriers would be established through different cohorts of EtoL changes accompanied by the downregulation of L1 transcription from these EtoL domains in the genome.

A surprising finding might be relevant to the putative link between upregulated L1s and replication timing features: the replication timing profile of human ESCs (hESCs), derived from preimplantation blastocysts, resembles the profile of more mature mouse EpiSCs, derived from the epiblast of post-implantation embryos, but not of mouse ESCs (mESCs) [49]. Mouse EpiSCs can be characterized as cells in which many EtoL domain changes are completed, and compact chromatin is accumulated near the nuclear envelope [46]. Therefore, a larger portion of the genome is likely to be represented by euchromatin in mESCs than in hESCs. This can be explained by the fact that FL-L1s are ten-fold more abundant in the mouse compared to the human genome [61]. It is reasonable to speculate that the number of upregulated FL-L1 units per genome might also be larger in mESCs than in hESCs. This could result in the abundance of early S replicating domains in mESCs, but not in hESCs, and lead to the euchromatinization of a larger portion of the genome in mESCs when compared with hESCs.

An aberrant execution of the developmental program is thought to be an important constituent of carcinogenesis [62]. The characteristic features of replication timing profiles of cancerous cells support this view. Findings in malignant cells from patients with acute lymphoblastic leukemia show that (i) replication-timing changes occur in units of the same size range (400–800 kb) as normal developmentally regulated replication domains; (ii) more than half of these changes align with the boundaries of developmentally regulated replication domains; and (iii) distinct replication timing changes can be considered a “pan-leukemic fingerprint”, which slightly overlaps with a “pluripotent fingerprint” [63]. An overlap of the replication timing profiles of another type of malignant cells, teratocarcinoma cells, and pluripotent embryonic cells can be even more profound. Teratocarcinoma cells that resemble embryonal carcinoma cells as well as cells of the ICM [64] are known to develop into normal tissues and germ line cells after transplantation to the blastocyst [65]. This suggests that the transplanted teratocarcinoma cells establish the same “pluripotent” replication timing and gene expression profiles as the recipient cells possess at the blastocyst stage. It is tempting to speculate that this can be achieved, at least in part, due to a similarity between single-stranded FL-L1 transcription profiles of teratocarcinoma and the ICM cells. In fact, the L1Hs, L1PA1, and L1PA2 subfamilies equally contribute to L1 transcript profiles of human embryonal carcinoma and ESCs, whereas older subfamilies are differentially represented in these cells [57].

### Epigenetic repertoire of full-length L1 transcripts

Several recent studies have shown that FL-L1 transcripts and L1 RT are implicated in epigenetic regulation of numerous genes in normal embryonic development and also in tumorigenesis [26,57,66]. However, the nature of this epigenetic regulation and the involved molecular mechanism(s) are largely unexplored and invite numerous future investigations. First, little is known about whether the expressed subsets of FL-L1s, and the putative epigenetic role(s) they might have, change during development. Second, it is not clear whether the active expression of single-stranded FL-L1 RNAs regulates the state of chromatin, and, if so, whether it promotes euchromatinization or heterochromatinization. Finally, it is not known whether transcription of an FL-L1 element, FL-L1 RNA, or reverse transcription of this RNA regulates or modifies the chromatin state.

Although sequence profiles of transcribed FL-L1s and their changes during development are largely unknown, some data demonstrate that the transcription of distinct subsets of L1s is likely developmentally regulated and stage-specific. Different patterns of expression of FL-L1s have been found on the X chromosomes during early (day 0–4) compared to late (day 8–10) stages of differentiation of female mESCs [66]. The precise sequence composition of L1s transcribed from the active X chromosome (Xa) and the Xi, their localization on the chromosome map, and the epigenetic role they might play during early ESC differentiation remain unknown. During the late stages of differentiation, when transcription of L1s in the nucleus and from the Xa is globally reduced, transcription of L1s from the Xi is still detectable [66]. This transcription is thought to be bidirectional and play a role in the production of siRNAs that promote heterochromatinization in *cis* and thus downregulate neighboring genes that escaped Xist-based silencing [66]. Importantly, sense transcription of FL-L1s seems to prevail over the bidirectional transcription in ESCs, which then appears to largely shift to bidirectional transcription of L1s as the cells differentiate. This notion is supported by two findings. First, the frequency of small RNAs derived from L1 elements of T_F_ subfamily is two-fold higher on day 5 of mESC differentiation than on day 0 [66]. Second, the activity of the L1 sense promoter is markedly more prevalent than the activity of the antisense promoter in hESCs, which expresses 10 to 15 times more sense L1 RNA than in differentiated cells [57]. Together, these data favor the hypothesis that the L1 RNA profiles are developmentally regulated.

Unidirectional (sense) and bidirectional transcription of FL-L1s can coexist in a cell, and they likely play opposite epigenetic roles. Both types of transcription of L1s have been found in ESCs [56,57,66]. Bidirectional transcription from the L1 5′ UTR may contribute to silencing of a portion of the chromatin domains through siRNA-based mechanism in ESCs. At the same time, unidirectional transcription of another subset of FL-L1s might promote the euchromatinization in *cis* of a different cohort of domains.

Although no direct evidence demonstrates that sense transcription of FL-L1s is implicated in euchromatinization in ESCs, this type of transcription of FL-L1s is associated with euchromatinization in cancer cells. This is supported by the fact that RNAi-based downregulation of the expression of FL-L1s as well as the inhibition of RT in transformed cells causes the reprogramming of chromatin segments to a more compact state in their derivates [30,38]. Because unidirectional sense transcription of FL-L1s appears to shift to bidirectional transcription upon differentiation of ESCs, it is tempting to hypothesize that the epigenetic role of FL-L1 transcripts might change in development.

How FL-L1 RNAs direct or mediate changes of chromatin conformation and transcriptional activity of neighboring genes is largely unknown. There are at least two potential types of FL-L1 transcripts in the nucleus — assembled and unassembled with ORF1p/ORF2p — that might have different epigenetic roles and underlying mechanisms. Thus, the sense transcription of an FL-L1 element and/or the transcripts, incorporated in *cis* into the chromatin, are essential for the formation and function of a neocentromere and the selective repression of genes within or adjacent to this domain [67]. It remains to be determined whether these transcripts are assembled with ORF1p/ORF2p or not and whether the sense transcription of FL-L1s inhibits the activity of neighboring genes in other genomic locations. FL-L1s, which form L1 RNP complexes with ORF1p and ORF2p in the cytoplasm [68-70], are found in ESCs and many cancer cell lines (discussed below). Upon entering the nucleus, such FL-L1 RNPs might drive a reverse-transcription-based mechanism linked to the establishment of a totipotent/pluripotent state in embryos and an undifferentiated state in many cancers. This idea is supported by findings from the FL-L1 knockdown and RT inhibition experiments discussed above. The results of these experiments also favor the idea that both transcription and reverse transcription of FL-L1s are integral steps of an unknown epigenetic mechanism. L1-encoded proteins preferentially associate with and act on L1 RNA, from which they are translated (a phenomenon termed *cis*-preference) [13,71,72]. Therefore, it is unlikely that RNAi-based knockdown of transcription of FL-L1s and the inhibition of L1-encoded RT could target separate epigenetic mechanisms and result in the same outcome. In this context, the question arises as to what part L1 reverse transcription plays in this mechanism.

Massive L1-linked reverse transcription found in mouse zygotic pronuclei and nuclei of the two-cell embryo is believed to be DNA replication independent for two reasons: (i) the exposure of the zygotes to aphidicolin, an inhibitor of DNA polymerase, 4 h after fertilization did not block DNA synthesis as evidenced by a significant incorporation of 5-bromodeoxyuridine (BrdU), the analogue of thymidine; however, when aphidicolin was used in conjunction with abacavir, a nucleoside inhibitor of reverse transcription, the incorporation of BrdU was strongly inhibited; and (ii) this aphidicolin-resistant abacavir-sensitive synthesis of DNA is observed 4–8 h after fertilization, whereas, according to older publications, DNA replication is thought to start 8–12 h post-fertilization [27]. The first point, namely the interpretation of aphidicolin-resistant synthesis of DNA as unrelated to genomic DNA replication, is based on the current concept of DNA replication that implies genomic DNA is replicated (with the exception to telomeres) solely by DNA-directed DNA polymerases [reviewed in [73]]. However, it is worth mentioning that the current concept of DNA replication, being well-established through numerous experiments and entrenched in the minds of the scientific community, has not been tested in all genome locations in all cell systems in all organisms at all possible conditions. Potentially unexplored exceptions to the well-known mechanism may exist in distinct genome locations and cell systems. Telomerase is a notable example of a reverse transcriptase carrying its own RNA molecule, which is used as a template to elongate chromosome ends [74]. L1 RNP could be another example of an enzyme-RNA molecular machinery driving genome-wide replication of L1 sequences. As research has progressed, it has become apparent that L1 RT and telomerase have remarkable similarities [[75] and references therein]. The second point with respect to DNA replication starting in the zygote 8–12 h after fertilization could be fallacious. The references provided by Vitullo and co-authors [27], when traced back to original publications, lead to results obtained by microdensitometry of Feulgen stained pronuclei [76], a low sensitivity methodology. The provided references also lead to publications in which dating of post-fertilization events was inferred, probably incorrectly, from time passed after the injection of human chorionic gonadotropin (HCG) [77,78]. More accurate estimations of the timing of pronuclear DNA synthesis in naturally ovulated and fertilized mouse eggs of six different genotypes, performed by cytofluorometric measurement of ethidium bromide-stained DNA, have indicated that the S phase starts at ~4 (3.8–4.6) h post-conception and lasts between 6.4 and 11.1 h in various genotypes [79]. Accordingly, it is reasonable to assume that the onset of ^3^H-thymidine incorporation in the pronuclei at 21 h post-HCG, which is thought to correspond to 7–9 h post-fertilization [77], and the onset of labeling with BrdU at 4 hr after fertilization [27] can be attributed to the same event: reverse transcription. The similarity of the early labeling patterns by ^3^H-thymidine and BrdU in male and female pronuclei [27,77] supports this notion. It is also worth noting that the incorporation of either ^3^H-thymidine or BrdU can only be interpreted as DNA synthesis but not as a particular mechanism thereof.

The DNA synthesis by reverse transcription found at the onset of mouse embryogenesis is thought to be L1-linked [27]. Data obtained by quantitative PCR (qPCR) analyses with primers designed to amplify FL-L1s of the T_F_ subfamily of L1s demonstrate an approximate two-fold increase of the L1 DNA copy number per haploid genome in the mouse zygote, two-cell embryo, and morula [27]; however, the time window and the phase of the cell cycle in which the qPCR analyses were performed were not indicated. Consequently, the design of the above-mentioned experiments [27] has led to results that are inconclusive in terms of whether the L1-linked DNA synthesis by reverse transcription is DNA replication dependent or independent.

In this regard, it is important to compare L1-related qPCR data obtained at two points of the zygotic cell cycle. The first point should be during the phase of the cell cycle when reverse transcription occurs but DNA polymerase-dependent DNA replication has not yet started. The second point should be when DNA replication is complete (i.e., in G2/mitosis). Although the results of such an experiment cannot provide evidence of the nature of the observed L1-linked DNA synthesis by reverse transcription (a potential synthesis of extragenomic L1 DNA copies cannot be ruled out), this approach could be a good starting point to test whether this reverse transcription is DNA replication dependent or independent. Therefore, the data available at this time are not convincing evidence of the massive nuclear reverse transcription occurring in early embryos being DNA replication independent.

### Hypothesis and rationale: two modes of L1 DNA replication as an epigenetic switch

**Figure 1:**
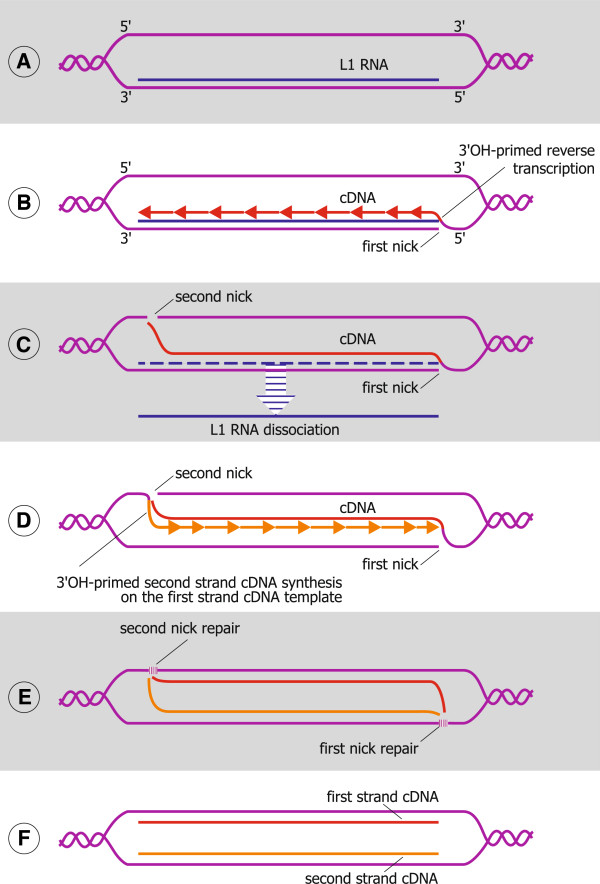
**A hypothetical mechanism of noncanonical L1 DNA replication. A**. Formation of an L1 DNA:RNA duplex. Heterogeneous FL-L1 RNAs, being assembled with L1 ORF1p and ORF2p, find their “parental” complementary sequences in the genome and form L1 DNA:RNA hybrids. The chaperone activity of ORF1p, which includes the melting of mismatched duplexes, is deemed indispensable for pairing of the L1 RNA with the fully complementary L1 DNA. The displaced DNA strand of an L1 unit is likely stabilized by auxiliary factors. **B**. First-strand cDNA synthesis. ORF2p bound to the 3′ end of the FL-L1 RNA nicks the bottom DNA strand and synthesizes the first cDNA strand from the liberated 3′-hydroxyl. **C**. Second nick formation. When ORF2p reaches the 5′ end of the L1 RNA, it nicks the top DNA strand at the 5′ end of the L1 element. ORF2p then switches templates from the RNA to the cDNA. The L1 RNA likely dissociates at this point. **D**. Second-strand cDNA synthesis on the first cDNA template. **E**. Nicking at the genomic DNA-cDNA junctions and the ligation of the segments of the “parental” DNA at the sites of the first and second nicks by auxiliary factors. **F**. Unpairing of the new L1 cDNA strands and their pairing with the “parental” strands by auxiliary factors. The ends of the new cDNA strands are joined with the new strands synthesized by the canonical mechanism on the adjacent segments of the “parental” strands. Each cell division produces two cells with equal amounts of old and new DNA synthesized by a combination of two different mechanisms.

In this review, I would like to propose that the L1-linked reverse transcription-based DNA synthesis found in early embryos and also likely to be found in undifferentiated cancer cells is part of the DNA replication program in these types of cells. This implies that two different mechanisms of DNA replication, canonical and noncanonical, can co-exist to replicate the genome during the same round of DNA replication in early embryos, ESCs, and many cancers. In these cell systems, a portion of genomic FL-L1 sequences is proposed to replicate by the noncanonical mechanism (i.e., L1 RNP-driven reverse transcription starting on an L1 RNA template bound to a complementary “parental” genomic L1 DNA sequence) (Figure 1). The noncanonical mechanism is proposed to trigger when FL-L1 RNAs are actively transcribed and translated and when full-size L1 RNPs are assembled. Full-size L1 RNP is herein defined as consisting of FL-L1 RNA, L1 ORF2p, and multiple trimers of L1 ORF1p (discussed below). Therefore, there can be two modes of DNA replication of FL-L1 sequences in the genome: canonical and noncanonical. The noncanonical mode of replication of FL-L1s is proposed to be L1 RT-driven, origin-independent DNA replication as a part of normal early development. The canonical (DNA polymerase-driven, origin-dependent) mode of L1 DNA replication is likely to replace the noncanonical replication in differentiating cells when the synthesis of full-size L1 RNPs is downregulated. The noncanonical mode of FL-L1 replication can be recapitulated in cancer cells.

**Figure 2:**
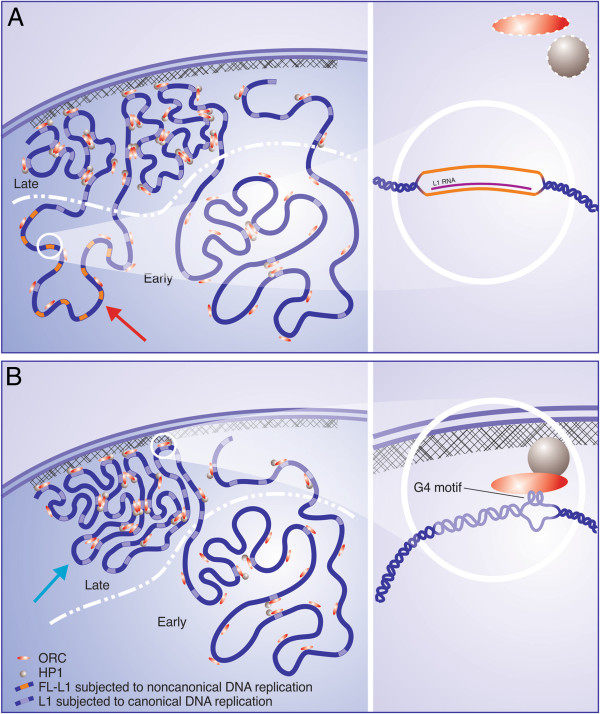
**A hypothetical model of two modes of replication of full-length L1s as an epigenetic switch. A**: Undifferentiated (early embryonic and cancer) cells. A subset of small L1-rich domains of the genome (EtoL domains as per Hiratani and co-authors [45]) replicates DNA in early S (red arrow). Transcriptional competence is imposed on these domains during early S phase of DNA replication. These L1-rich EtoL domains do not tether to the nuclear lamina and have a loose conformation of chromatin loops. Genes residing in these domains are linked to undifferentiated states of a cell. Noncanonical replication of FL-L1s residing in these domains might prevent them from binding to the nuclear matrix and from recruiting the ORCs (panel A, right) and, therefore, from being silenced through the ORC/HP1-mediated pathway of heterochromatin assembly. **B**: Differentiated and differentiating cells. Global programmed downregulation of FL-L1s upon differentiation of pluripotent embryonic cells results in switching “off” the noncanonical mechanism of L1 DNA replication. In the absence of L1 RNA paired with a complementary “parental” L1 DNA for noncanonical L1 DNA replication, L1s might attach to the nuclear matrix and form intrastrand G4 structures. The majority of replication origins are associated with G4s [80]. The L1-bound ORC might bind HP1 in a distinct chromatin environment and, thereby, play a crucial role in the establishment of a silent state on the L1-rich/gene-rich EtoL domains (panel B, right). These domains switch to their default state characterized by late replication, dense conformation, and tethering to the nuclear periphery [50] (blue arrow). Basically, the same switch from noncanonical to canonical replication of L1s residing within EtoL domains might occur upon differentiation of poorly differentiated cancer cells caused by the knockdown of the expression of L1s.

The next logical question is to why replication of FL-L1 sequences by either the canonical or the noncanonical mechanism is important for a cell. The answer could be that the switch from the noncanonical to canonical mode might be a fail-safe means to keep a large set of embryo-specific genes stably silent when the noncanonical mechanism of DNA replication is “off”. Specifically, the noncanonical mechanism of L1 DNA replication may serve as a noncanonical epigenetic determinant that regulates the transcriptional competence of a large cohort of neighboring genes. This regulation could be implemented through the prevention of a set of L1-rich EtoL domains from being tethered to the inner nuclear membrane (INM) and from being packaged into late-replicating facultative heterochromatin (Figure 2A). It follows then that when FL-L1 sequences are not replicated by the noncanonical mechanism, they would tend to be silenced due to their sequence composition. Sequence features of L1s might favor anchoring to the nuclear matrix and binding of the origin recognition complex (ORC) – two potential mechanisms that may contribute to silencing of L1s and adjacent sequences. The ORC might facilitate heterochromatin assembly and tethering of L1s to the nuclear periphery (discussed below). Therefore, origin-based replication of a distinct set of L1s might also be considered an epigenetic mechanism, which contributes to the default silencing of the involved domains (Figure 2B). The noncanonical replication of FL-L1s might exert rather specific, albeit different, effects on gene expression profiles depending on the subset of polymorphic FL-L1s involved in noncanonical DNA replication. This implies that a cell type-specific subset of noncanonically replicated FL-L1s determines the cohort of L1-rich EtoL domains that are transcriptionally competent in this particular type of cells.

A notable insight into the initiation of DNA replication in eukaryotic systems has brought about the concept of a “relaxed replicator” as a “context-dependent element”, which includes a DNA sequence in conjunction with DNA topology, DNA methylation, chromatin-bound proteins, transcriptional activity, and short-/long-distance chromatin effects [81]. This concept implies that the binding of the ORC to chromatin is guided by distinct combinations of sequences, chromatin contexts, and components of nuclear structure [81,82]. Accordingly, the replicator-initiator interactions are thought to have an additional function (or functions) beyond their role in DNA duplication [81]. In this context, it is logical to surmise that numerous ORCs, which remain bound to DNA by ORC2-5 subunits throughout the cell cycle [83], influence the formation of a certain chromatin environment through the recruitment of chromatin proteins and binding to the nuclear matrix. Indeed, a growing body of evidence indicates that the ORC is essential for the formation of heterochromatin in eukaryotes [84-87]. In mammals, the ORC recruits heterochromatin protein HP1 [86,87]. Factors that facilitate this process have begun to be revealed, one of which is an H3K9me3 environment [87].

In the context of nuclear structure, a significant portion of LINEs seem to be ORC-binding sites and function as MARs. This is suggested by the fact that origins colocalize with MARs [83,88,89] and that human LINEs are overrepresented among S/MARs, comprising 40% of the sequences [23]. The high overrepresentation of LINEs among S/MARs could be because S/MARs [90] and L1 sequences (discussed below) share a particular feature: partial unpairing of DNA strands. S/MARs are functionally heterogeneous; SARs are mainly transcription-linked, and MARs are replication origin/silent gene-associated [25,91]. Taking into consideration the functional heterogeneity of S/MARs and the tendency of L1-rich domains to be silent and replicate late at the nuclear periphery in differentiated cells [45,92], L1s can be even more over-represented among the origin-associated MARs than “bulk” S/MARs.

Several other facts also support the notion that the sequence composition of L1s makes them prone to bind ORCs. For example, poly(dA:dT) elements (5 mers or longer tracts), known to be present within L1s, disfavor nucleosome occupancy not only over themselves but also over adjacent regions [93]. Low nucleosome occupancy is thought to be a necessary, but not sufficient, requirement for the assembly of ORCs and pre-replication complexes near these regions [94]. Another feature of L1 sequences that might be favorable for ORC binding is a guanine-rich tract known to form an intrastrand tetraplex (G-quadruplex or G4) in the L1 3′ UTR [95]. This feature is present in all L1s with intact 3′ UTRs [95] and conserved throughout mammalian evolution [96]. About 90% of human origins are represented by G4-forming motifs [80], and these structures are known to be nucleosome-free regions [97]. Taken together, these data suggest that ORCs are highly likely to bind to G4 structures of those L1s that tether to the nuclear matrix.

If the sequence features of L1s (G4 structures, the tendency for partial unwinding, and nucleosome disfavoring) do promote ORC binding, the L1-bound ORCs may be essential for establishing a very stable silent state on L1-rich segments of the genome. One potential mechanism of the ORC-dependent silencing of L1s could be the recruitment of HP1 to the L1-bound ORCs. HP1γ, one of the isotypes of HP1 associated with the foci of facultative heterochromatin [98], is known to contribute to the silencing of FL-L1s [99]. Knockdown of the Cbx3 gene that encodes HP1γ activates repressed L1s [99]. The strong binding activity of HP1γ with lamin B receptor, an integral protein of the INM [100,101], could also be involved in the sequestration of L1s to the nuclear periphery. The recruitment of HP1 to the ORC is guided by H3K9me3 [87]. Although H3K9me3 is weakly represented on L1 sequences regardless of whether L1s are active or silent [102-104], H3K9me3 is overrepresented within L1-rich dark (Q or G) bands [105]. Therefore, it would be timely to gain insight into the putative link between the ORC, HP1, and H3K9me3 with regard to L1 silencing.

Another potential mediator of the ORC-dependent silencing of L1s might be an ORC-binding factor ORCA (ORC-associated protein). ORCA associates with the ORC in the presence of repressive histone marks and methylated DNA and functions as a facilitator of heterochromatin formation [87]. Thus, although it is not completely understood how a very stable silent state is imposed on L1-rich domains, G4-forming motifs within L1 sequences might be ‘landing pads’ for the ORC, the important player in heterochromatin assembly.

A genome-wide origin mapping study in hESCs and embryonic fibroblasts [80] has contributed a very important finding by demonstrating that EtoL developmentally regulated replication domains acquire some additional origins when they switch their replication timing from early to late S phase. Despite a general positive correlation of early replication with the high density and frequency of the usage of origins, the EtoL replication domains had even slightly lower origin scores when they were early replicating than when late replicating [80]. The exact localization of origins on the sequences of EtoL domains could clarify whether the additional origins accuired upon the EtoL transition during differentiation of pluripotent hESCs are L1-associated.

Together, these facts favor the hypothesis that L1s within developmentally regulated EtoL domains can be points of a strong attachment to the INM and peripheral nuclear matrix, thus keeping these domains in the default silent state. Importantly, such a role may be linked to the binding of the ORC by G4 within the L1 3′ UTR and, therefore, to canonical origin-based replication. L1 RNP, the molecular machinery of the proposed noncanonical L1 DNA replication, could be a more successful competitor for L1 sequences than the nuclear matrix and the ORC, which would preclude the L1 silencing scenario. Undoubtedly, L1-MAR and L1-ORC relationships need to be investigated in differentiated and non-differentiated cell systems and viewed in the context of developmentally regulated replication domains.

Relevant to this discussion, are three important points. First, experimental tethering of a number of loci to the INM causes their downregulation and the repression of neighboring genes and genes that are located far from the loci. However, experimental untethering by using a competitor compound that binds the target site induces the repositioning of the locus and adjoining segments away from the nuclear periphery and re-establishes transcriptional competence [106]. Second, transcriptional competence is established at the time of replication [107]. Early S replicating sequences are assembled into nucleosomes enriched with acetylated histones H3 and H4, the marks of open chromatin, as opposed to late S replicating DNA, which is packaged mainly into silent chromatin marked by deacetylated forms of these same histones [107,108]. Third, the nuclear periphery, which is essentially a repressive environment, has early S replicating and transcriptionally active subcompartments [59,109-111] that appear to be more prominent in early embryonic and transformed cells than in differentiated cells.

Taking all of this into account, it can be speculated that L1s, being untethered from the INM and repositioned into early S replication compartments, could then assemble with acetylated H3/H4. Indeed, activation of L1s in HeLa cells by a carcinogen, benzo(a)pyrene, increases the H3K9ac mark at the L1 5′ UTR [104]. As proposed above, L1 RNP bound to complementary L1 DNA and/or noncanonical L1 DNA replication might favor the untethering of the implicated chromatin domains from the INM. These liberated segments can relocate to the nuclear interior, the location of dominating early S replication [112] and transcriptional competence. Alternatively, these liberated domains can become early S replicating and transcriptionally competent without noticeable repositioning towards the nuclear interior. This idea is consistent with the observation that the nuclear periphery can be almost entirely (mouse zygote) or partially (mESCs, many types of cancer cells) represented by euchromatin [58,59,113], which appears to replicate in early S phase, at least in the zygote [59]. In ESCs, the small size of alternating early- and late-replicating domains, together with the anchorage of late S-replicating segments to the INM [45], suggest that many small L1-rich early S-replicating pluripotency “indicator” domains are restrained in the nuclear periphery. The localization of L1-encoded proteins within the nucleus can be a cue to where L1 RNPs may act with regard to the nuclear periphery. In A-375 melanoma cells, L1 ORF2p-specific fluorescent signals appear as a dense rim in the nuclear periphery and patches of sparse speckles that protrude into the nuclear interior [30]. However, in the colon cancer cell line H1299, ORF1p-specific signals form multiple foci across the entire space of the nucleus [114]. This suggests that L1 RNPs may act in the nuclear periphery and in the nuclear interior in a cell type-specific manner.

A replication-timing program, which governs the transcriptional competence of chromosome domains, is established during early G1 phase, a short window of opportunity termed the timing decision point (TDP) [115]. Post-mitotic re-establishment of 3D chromatin architecture occurs at the TDP, and developmental cues that change a replication-timing program are likely to act during this short time window [115]. If the proposed noncanonical replication of L1s does occur and function as a regulator of replication timing and spatial positioning of the involved domains, L1 RNA-L1 DNA interactions for DNA replication should be established no later than the TDP. This means that the sites of noncanonical DNA replication are likely to be licensed from early G1 onward, and their licensing could serve as an epigenetic determinant. Alternatively, this epigenetic role could be performed by noncanonical replication of L1s if it starts at the TDP. The latter could be the case during the first round of DNA replication in the embryo. Noticeable DNA synthesis by reverse transcription, which precedes DNA polymerase-dependent DNA replication in mouse pronuclei [27], could be the first phase of DNA replication and serve as the epigenetic mechanism implicated in the establishment of the initial replication timing program and chromatin architecture. From this viewpoint, it is not surprising that the DNA synthesis by reverse transcription is more prominent in the male than the female pronucleus [27] because the hypercondensed paternal chromatin requires more extensive reorganization than the maternal chromatin. The organization of sperm chromatin favors the early onset of L1-related reverse transcription in the male pronucleus. Specifically, a small portion of the genome is undermethylated and packaged with histones into active nuclease-hypersensitive chromatin; these segments of the genome are highly enriched with L1s [116,117]. These L1 sequences are found at the periphery of the sperm nucleus [116], the same location where pronuclear reverse transcription occurs [27].

### Biological significance of L1 RNP: a step beyond retrotransposition

Two L1-encoded proteins, ORF1p and ORF2p, are translated in unequal amounts from a bicistronic FL-L1 transcript [1,118] and bind to the RNA from which they are translated [1,13,72]. This suggests that L1 RNP functions as a molecular machinery *in vivo*. ORF1p forms trimers that polymerize under the very conditions that support high-affinity nucleic acid binding [119]. Polymerized trimers of ORF1p bind to L1 RNA, and one or two molecules of ORF2p attach at or near the L1 RNA poly(A) tail [1,13,72,119]. ORF1p possesses a nucleic acid chaperone activity on oligonucleotide substrates *in vitro*; specifically, it promotes accelerated and stringent annealing of complementary nucleic acid sequences by facilitating the melting of imperfect duplexes, strand exchange, and the stabilization of perfect duplexes [120,121]. However, the biological significance of the ORF1p chaperone function is poorly understood.

First, it is unclear what type(s) of duplexes ORF1p promotes the formation of *in vivo*. On one hand, the chaperone function of ORF1p has been demonstrated on DNA oligonucleotides in *in vitro* assays [120]; on the other hand, ORF1p preferentially binds to L1 RNA *in vivo* and *in vitro*[69,122]. Considering the complementarity of the poly(A) tail of L1 RNA to the poly(T) segment of a typical 5′ T_n_/A_n_ 3′ cleavage site of L1 EN [123], formation of a short DNA:RNA duplex is proposed to occur to prime reverse transcription during retrotransposition [120]. ORF1p is also speculated to promote the exchange of a DNA:DNA duplex to an RNA:DNA hybrid at the target site [120]. However, the enormous mass of ORF1p trimers that bind to L1 RNA [121] seems excessive to merely promote the formation of a short RNA:DNA duplex to prime cDNA synthesis *in vivo*. Moreover, because the liberation of 3′-OH at the nick site is sufficient to prime reverse transcription on an L1 RNA template *in vitro*[124], it remains uncertain whether such short RNA:DNA duplexes are indeed formed to initiate reverse transcription *in vivo*.

Second, it is unclear what processes require ORF1p as a chaperone *in vivo*. Its implication in retrotransposition might not be the only role it plays. Endogenous L1 RNAs, which form L1 RNPs in hESCs, belong not only to retrotranspositionally active (L1Hs) but also to retrotranspositionally inactive L1 subfamilies (L1PA2, L1PA3, L1PA4, L1PA6, and L1PA7) [56]. It is unlikely that hESCs synthesize retrotransposition inactive L1 RNPs having no function. Therefore, ORF1p as a part of retrotranspositionally inactive L1 RNP might play a yet unknown role.

ORF1p is deemed essential for the retrotransposition of L1s expressed from L1 constructs in transfected cells [121,125,126]. This is evidenced by the fact that mutant ORF1 proteins with impaired chaperone function but unaffected RNA-binding activity abolished or reduced retrotransposition in comparison with the wild-type (wt) ORF1p in cell-based assays [125]. Although ORF1p is non-essential for retrotransposition in a cell-free *in vitro* assay [124], its availability increases the quantity and length of nascent cDNAs and promotes the initiation of cDNA synthesis at more typical retrotransposition start sites [72]. The role of a non-mutant ORF1p in the retrotransposition of a “synthetic” L1 element in cell-based assays might be the same as in a cell-free system. Specifically, it could promote the synthesis of a longer cDNA strand, including a reporter cassette upstream of the L1 3′ UTR, so that a retrotransposition event is detectable.

While the integration of a “synthetic” L1 element into the genome is random [127], the integration of endogenous L1s seems to be non-random and biased to a similar sequence environment. Although post-insertional selection and recombination influence the genomic distribution of L1s, the non-random integration of endogenous L1s appears to be an important factor in the biased localization of L1s in GC-poor/AT-rich regions of the genome [128-132]. Analyses of the distribution of L1s in mammalian genomes have led to the conclusion that L1s tend to cluster [130,133]. However, there is no current consensus on whether clustering is a general feature of L1s [130] or more pronounced among old L1 elements [133]. The 100 kb flanking sequences of human L1s of a currently active subfamily Ta-1 (also known as L1Hs-Ta1) and older L1s (L1PA2 and L1PA5) are enriched in L1 DNA [130]. Interestingly, the sex chromosomes, which are enriched in ancestral L1s, are much less hospitable for Ta-1 insertions than chromosome 4, which is enriched in Ta-1 elements [130]. Although L1s are estimated to insert in pre-existing L1s only 13% of the time [134], the portion of L1-derived sequences that harbor new L1 insertions can be larger. Remains of the 3′ polyA tails of previous L1 insertions that bear L1 EN recognition motifs are thought to be common target sites for L1 retrotransposition [123].

Despite the incompleteness of our knowledge regarding the incidence, degree, and length of sequence similarity between L1 insertions and surrounding regions, available data fit the concept of sectorial mutagenesis introduced by Jurka and Kapitonov [128]. This concept implies that new insertions of transposable elements tend to occur in specific chromosomal regions. Importantly, the density of LINEs correlates more strongly with specific orthologous segments of the human and mouse genomes than with the local GC content [3].

The factors that determine the non-random integration of endogenous L1s and random insertions of “synthetic” L1 elements remain unexplored. It has been hypothesized that the higher frequency of target sites and the open state of chromatin could contribute to the insertional bias of endogenous L1s [132]. The fact that “synthetic” and endogenous L1s target the same consensus sequence [127,134], but demonstrate different patterns of retrotransposition, does not favor the notion that the frequency of the target sites could be a key factor of non-random retrotransposition of endogenous L1s. The open state of chromatin established on certain chromosomal domains might be a favorable condition rather than a determinative factor for non-random retrotransposition of L1s.

Not excluding other factors that can contribute to the insertional bias of L1s, I hypothesize that retrotransposition of endogenous L1s might be linked, at least to some extent, to noncanonical DNA replication. This may cause non-random retrotransposition of endogenous L1s if this process fails. More random retrotransposition of “synthetic” L1s might be caused by the inability of a reporter cassette bearing L1 RNA to pair with a complementary sequence in the genome to perform noncanonical DNA replication. Moreover, the chaperone activity of ORF1p might be essential for the recognition of complementary genome sequences by L1 RNAs and their pairing. ORF1p that promotes the melting of imperfect duplexes may contribute to random retrotransposition of “synthetic” and non-random retrotransposition of endogenous L1s. In addition to the known biased insertions of L1s, other findings discussed below favor this hypothesis.

An unequal potency of retrotransposition among endogenous FL-L1s capable of producing functional proteins is thought to be, at least in part, due to differences in some measures of the chaperone activities of ORF1p variants [121]. Importantly, a reference point on the scale of retrotransposition potency, also often termed as wt, can, paradoxically, be a measure of the failure of distinct ORF1 proteins to perform other biologically essential functions. An example of such an L1 element in mice could be a retrotransposition-efficient variant of L1_spa_ that encodes ORF1p with an aspartic acid codon at residue 159 (D159) [121]. In contrast to the D159 variant, another variant of L1_spa_ that encodes ORF1p with a histidine codon (H159) at this position is known as a retrotransposition-inefficient element [121]. Interestingly, the less active variant, H159 ORF1p, is much more successful at melting a mispaired DNA duplex than the more active D159 ORF1p, which is not able to fully melt an imperfect duplex in the absence of strand exchange [121,126]. If L1 RNP does perform an important function on genomic DNA that requires perfect pairing of L1 RNA and complementary DNA, the efficient melting of mismatched duplexes by ORF1p could be essential for displacing L1 RNA from a mispaired DNA:L1 RNA hybrid and, therefore, for promoting the formation of completely paired L1 DNA:RNA hybrids. Consequently, L1 RNA that encodes ORF1p capable of efficient melting of mismatched duplexes might be less prone to retrotransposition *in vivo*.

Sequence composition of L1s favors the formation of L1 DNA:RNA hybrids reminiscent of long R loops. An R loop is an unwound DNA segment, one strand of which associates with the complementary RNA, whereas the second DNA strand appears as a displaced loop [135]. A/T richness and paired stretches of polypurines:polypyrimidines, the characteristic features of L1s, are required for dsDNA to be prone to the formation of an R loop [135]. The formation of R loops spanning several kb is possible; however, auxiliary factors are required for the unwinding and stabilization of long ssDNA segments [135].

If the noncanonical mechanism of DNA replication does exist and new integration events of L1s are indeed linked to their noncanonical replication in early embryos and certain types of cancer, then L1 retrotransposition can be expected to occur in these cell systems rather than in all types of cells where L1s are actively transcribed. It has become evident that retrotransposition of genomic L1 elements occurs mainly in early embryonic cells but not in germline cells, as previously thought [19,136], and in certain types of cancer cells but not in normal tissue counterparts [20,21]. Despite the fact that L1 RNA is available in female germ cells and tremendously abundant in spermatogenic cell fractions, retrotransposition events are rare in the germlines; this is in contrast to much more frequent integration events in preimplantation embryos [19]. Interestingly, L1 RNA that is retrotransposition inactive in the germlines is carried over into the embryo where it remains stable and then becomes retrotranspositionally active in the cleaving embryo [19]. L1 RNA transcribed in the embryo causes even more retrotransposition events than does carried-over L1 RNA [19]. Direct evidence of endogenous L1 retrotransposition associated with L1 activation in cancer cells has recently been reported [20,21]. In transgenic mice carrying the human L1 element, retrotransposition events have been found to occur in chemically induced skin tumors but not in the adjacent normal skin tissue [20]. As shown by two high-throughput L1-targeted resequencing methods, retrotransposition of L1Hs occurs in certain human colorectal tumors but not in the surrounding normal colon tissues [21]. Importantly, the number of new L1 insertions in human colorectal tumors was not correlated with the degree of hypomethylation of L1 promoters [21]. These findings suggest that the activation of L1 expression as a result of L1 demethylation is a necessary but not sufficient condition to cause a high retrotransposition rate.

Further investigation of some identified hotspots of L1 insertions is required to determine conditions and molecular processes that might favor L1 retrotransposition on the genome scale. Such hotspots have been found in the vicinity of certain genes expressed in gonads and during embryogenesis [132]. If retrotransposition of L1s is linked to their noncanonical replication, L1 insertions are anticipated to be biased to certain sets of L1-rich EtoL domains, the early replication and transcriptional competence of which is characteristic of either embryonic or cancer cells. In this context, it would be interesting to study two potential links regarding the L1 integration hotspots: (i) the link between the L1 sequences integrated within the hotspots and FL-L1 RNA species carried over into the zygote and expressed during development, and (ii) the link between these hotspots and L1-rich EtoL developmentally regulated domains.

The functional features of L1 ORF2p could potentially make it capable of providing the putative noncanonical replication of FL-L1 loci. In *in vitro* assays, L1 RT has demonstrated a high processivity on both RNA and ssDNA templates and the ability to switch templates from RNA to cDNA in order to synthesize the second strand cDNA [124,137]. This is consistent with the capability of L1s to generate full-length insertions *in vivo*. L1 EN generates single strand nicks in dsDNA with a preference for TA dinucleotides within 5′ TTTT/AA 3′ target tracts; additionally, L1 EN is able to efficiently nick other sets of dinucleotides within a loose consensus sequence [123,138]. From the perspective of the proposed model, this nicking flexibility might be essential to generate two nicks in order to prime first and second strand cDNA synthesis. The first nick might occur in the bottom strand complementary to an L1 A-rich tail, which is known to consist of the AATAAA polyA signal followed by A_n_ interrupted by short GT- or T-rich motifs [139]. A putative location of the second nick could be in the top strand at the beginning of the L1 5′ UTR. An interesting nuance is that L1 EN activity increases dramatically on an unwound DNA helix [123].

Together, these findings favor the hypothesis that L1 RNP functions as the molecular machinery of noncanonical replication of L1 units in concert with other cellular factors that are likely to be available when this mechanism is active. Both L1-encoded proteins appear to be indispensable for the proposed mechanism, and this implies that only those FL-L1 transcripts that are assembled with both proteins can function in terms of noncanonical replication. The strong preferential binding of ORF1p and ORF2p with their encoding L1 RNA and the chaperone activity of ORF1p can provide a high level of specificity in recognition of “parental” L1 DNA units subjected to noncanonical replication and, therefore, in epigenetic targeting on a genomic scale.

### L1 RNA and proteins: what, where, and when?

To better understand the epigenetic role(s) of the activated FL-L1s, it is important to determine the patterns of L1 transcription and synthesis of L1 proteins in different types of cells and potential links between these patterns and cell phenotypes. It has long been accepted that the production of FL-L1 RNAs occurs notably in the germline, early embryos, and many types of cancer cells, whereas it is mainly shut down in the majority of normal unstressed somatic tissues. However, a highly complex picture of L1-involving pathways in a tissues-specific context has started to emerge. First, recent research shows that cells from a broad range of normal organs actively synthesize FL-L1 RNAs [140]; however, the majority of these transcripts undergo splicing and/or premature polyadenylation [140-142]. Unfortunately, the scant amount of data on L1 RNA sequences and proteins in some organs, e.g., in the placenta and esophagus [140,143,144], does not allow for a definite conclusion on these patterns. Second, there is some uncertainty regarding the interpretation of data obtained by methodological approaches appropriate for a less complex system. (For a discussion of these issues, please see Additional file 1). Consequently, the tissue specificity of the synthesis of FL-L1 RNAs and full-size L1 RNPs and correlations with distinct phenotypic features can be discussed only with a limited degree of confidence. Third, there appears to be different patterns of expression of FL-L1 RNAs that might not necessarily result in the production of full-size L1 RNPs. Therefore, the relationship between the synthesis of FL-L1s and phenotypic properties might not be straightforward. Finally, the assembling/functioning of full-size L1 RNPs seems to be suppressed in gametes, but becomes activated after fertilization.

**Figure 3:**
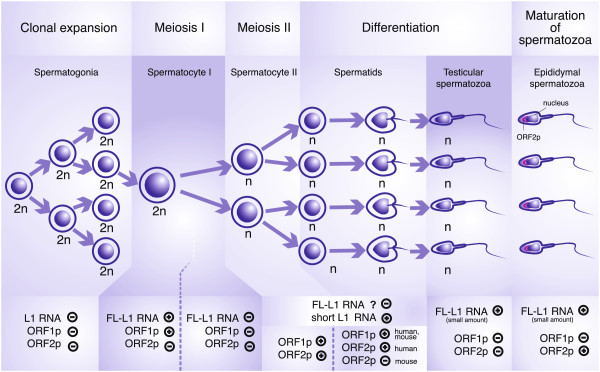
**Patterns of expression of L1 RNA, ORF1p, and ORF2p during spermatogenesis.** Available data suggest that the synthesis of full-size L1 RNP consisting of FL-L1 RNA, ORF1p, and ORF2p is repressed during spermatogenesis. No L1-related products have been found in spermatogonia. Transient expression of FL-L1 RNA and ORF1p (but not ORF2p) occurs in spermatocyte I at the onset of meiosis (leptotene through mid-pachytene stage of prophase I). These upregulated FL-L1s are implicated in chromosome pairing [145]. Transcription of L1s resumes in spermatocyte II and continues in spermatids. However, L1 RNAs are mostly short, spliced, and prematurely polyadenylated species that translate into either ORF1p or ORF2p. Their functional significance is not known. Transcription of L1s, ORF1p, and ORF2p is downregulated by the spermatozoa stage. A small amount of FL-L1 RNA (not detectable by Northern blotting) is available in spermatozoa. ORF2p, not found in testicular spermatozoa [18], is detectable in the sub-acrosomal space in mature sperm [27].

Currently, there is no convincing evidence that noticeable amounts of full-size L1 RNPs are synthesized in either male (adult and prepubertal) or female germline cells. Available data suggest that either FL-L1 RNA and ORF1p are synthesized, but ORF2p is missing (as observed in early meiotic spermatocytes) or present at a very low level (female gametes), or L1 proteins are produced from shortened L1 transcripts (as observed in secondary spermatocytes and spermatids) (Figure 3). Therefore, execution of an RT-mediated program might be blocked in germline cells. Specifically, in adult human testes, L1-related poly(A) RNAs are extremely abundant; however, no FL-L1 RNA has been found by Northern blotting because the majority of FL-L1 RNAs undergo premature polyadenylation combined with splicing [140-142]. These processed L1 RNAs can potentially translate into either ORF1p or ORF2p or their truncated forms. Indeed, both ORF1p and ORF2p (or their truncated forms, as discussed in Additional file 1) have been detected by immunostaining in somatic testicular cells, secondary spermatocytes, and immature spermatids in adult human testes [143]. Similarly, no FL-L1 RNA has been detected in adult mouse testes by Northern blotting, whereas short L1 transcripts of variable lengths were abundant in both germ and somatic cells [18]. In adult mouse testes, ORF1p-related immunostaining has been detected in somatic cells and spermatids, but no ORF2p-specific immunostaining has been revealed [18].

Although processed L1 transcripts prevail in adult testes, FL-L1 RNAs, which are undetectable by Northern blotting, might be present in early meiotic (leptotene and zygotene) spermatocytes. This cell fraction is rare in adult mouse testes but is much better represented in prepubertal testes where it accounts for the abundant ~7 kb sense-strand L1 transcripts [18]. The transient expression of L1s and ORF1p coupled with L1 DNA demethylation is intrinsic to the onset of normal meiosis (leptotene through mid-pachytene stage) in every round of spermatogenesis [145,146]. This type of L1 expression, downregulated in late meiotic prophase I, is unrelated to the production of processed L1 transcripts triggered later in spermatogenesis [18,145]. The transient expression of FL-L1s and ORF1p is proposed to be a programmed, though not understood, event associated with chromosome pairing and assembly of the synaptonemal complexes in male meiosis [145]. Because L1 retrotransposition is highly repressed in the germline compared with early embryogenesis [19], and ORF2p appears to be unavailable in early spermatocytes, it can be speculated that the L1 RNPs, implicated in early male meiosis, are not full-size L1 RNPs. Similar to the onset of male meiosis, ORF1p is transiently expressed in female germ cells entering meiotic prophase I in the mouse embryonic ovary [144], suggesting the same role of L1 expression in chromosome pairing.

Another category of germ cells likely to accumulate small amounts of FL-L1 RNA are male and female gametes. This is supported by the fact that FL-L1 RNA carried over into the zygote by both gametes causes detectable retrotransposition events in the embryo [19]. Because the carried-over FL-L1 RNA remains stable and capable of retrotransposition during early embryogenesis [19], it might be implicated in the L1-linked RT-dependent synthesis of DNA not only in the zygote but also in the cleaving embryo. While small amounts of FL-L1 RNA seem to be present in both gametes, it remains unexplored whether this RNA is assembled into RNP with one or both L1-encoded proteins. The synthesis of ORF2p is downregulated in testicular sperm cells [18] but appears to resume in the epididymal spermatozoa because ORF2p is found in the sub-acrosomal space of these cells [27]. Therefore, the synthesis of ORF2p seems to restart at the terminal stage of spermiogenesis when the synthesis of ORF1p is downregulated. As shown by immunostaining, ORF1p and ORF2p are barely detectable at the terminal stages of mouse oogenesis [27,144]. In a full-size L1 RNP, ORF1p is typically present in great excess compared to ORF2p [1]; therefore, weak ORF1p-specific immunostaining can reflect the downregulated synthesis of ORF1p in oocytes. Because of the paucity of ORF2p present in L1 RNP, [1], weak ORF2p-specific immunostaining dispersed within the cytoplasm of the oocyte [27] may not suggest the lack of ORF2p if compared with the amount of ORF2p in the epididymal spermatozoid. Together, these findings favor the assumption that small amounts of FL-L1 transcripts can be stored in both male and female gametes, but the formation of FL-L1 RNA/ORF1p/ORF2p complexes might be blocked due to the downregulated synthesis of ORF1p. ORF2p, which is synthesized at the very terminal stage of sperm maturation and also seems to be present in the oocyte, could be destined to initiate the synthesis of L1 DNA by means of reverse transcription in both zygotic pronuclei.

Preimplantation embryos likely synthesize full-size L1 RNPs; however, systematic studies of L1 RNAs/ORF1p/ORF2p are required for definite conclusions. Strongly upregulated expression of L1s [147], noticeable RT-dependent DNA synthesis, and the significant increase of L1 copy number in two-cell mouse embryos [27] suggest that L1 RNPs are likely present and function during this stage. The abundance of sense-strand FL-L1 transcripts in mouse blastocysts [14] and the presence of FL-L1 RNAs and ORF1p assembled into RNPs in hESCs and iPSCs [56,148] favor the idea that full-size L1 RNPs can be present at least in pluripotent cells of the blastocyst.

The exact developmental window when such RNPs are formed remains to be determined. Although genome-wide intense upregulation of L1s occurs and plays an important role in preimplantation embryos, the less apparent production of sense-strand FL-L1 RNAs and proteins can still be present or transiently reinstated in distinct lineages or cell types later in development. The possibility of L1 expression and retrotransposition in human neural progenitor cells is suggested by the increased copy number of endogenous L1s in adult brains when compared with heart and liver samples obtained from the same individuals [149]. Moreover, mouse myogenic precursors, the differentiation of which is promoted by nevirapine [38], could also be a cell type that synthesizes some amount of full-size L1 RNPs.

Several types of cancer cells also seem to synthesize full-size L1 RNPs. With regard to L1-related products, the most studied cancer cells are cell lines derived from germ-cell tumors, mostly testicular, that are embryonal carcinomas and teratocarcinomas (teratomas with an embryonal carcinoma component) [64]. Embryonal carcinoma cells are highly malignant counterparts of the ICM: they express pluripotency markers and can be maintained as undifferentiated cells or induced to differentiate by morphogens [64,150]. Mouse F9 and C44 embryonal carcinoma and human NTera2D1 teratocarcinoma cell lines are known to actively synthesize sense-strand FL-L1 RNAs [15,16]. These transcripts form RNPs with ORF1p in F9 and C44 cells [16,68]. The presence of RT activity associated with L1 RNPs in NTera2D1 cells [151] suggests that full-size L1 RNPs may be synthesized in these cells. The fact that the malignant pluripotent cells originate from germ cells but not other cell types could be explained by the proposition that L1 RNAs, synthesized during gametogenesis and carried over into the zygote, have a pluripotency-linked function in the early embryo.

FL-L1s and ORF1p are also upregulated in a range of tumors and transformed cell lines, and this upregulation correlates with a transition to undifferentiated phenotypes, higher tumor grade, and poorer prognosis [17,114,140,152-154]. Despite the lack of parallel analyses of ORF2p in many studied cancers, the results discussed in the second section of this review suggest that ORF2p is also present in numerous poorly differentiated tumors. Consequently, the synthesis of L1 RNPs is likely a characteristic feature of many cancers.

In addition to many types of cancers, the activation of L1s might be intrinsically linked to cell dedifferentiation in certain regenerating cell systems. For example, L1-like retrotransposon that encodes ORF1 and ORF2 is dramatically upregulated in the blastema during axolotl (*Ambystoma mexicanum*) limb regeneration [155]. This activation of L1s slightly precedes the upregulation of a limb regeneration marker [155]. Interestingly, the completion of the regeneration of the amputated limb was accompanied by a 16% increase in L1 DNA copy number [155]. Surprisingly, the second wave of regeneration after re-amputation of the same limb resulted in a 70% increase in L1 DNA copy number [155]. Although the nature of this enormous increase in L1 copy number is not known, the authors interpret their data as retrotransposition. It is tempting to speculate that the herein proposed noncanonical mechanism of L1 DNA replication might be recapitulated in blastema to allow cell dedifferentiation. Moreover, the increase in L1 DNA copy number after the completion of the regenerative process could be due to the accumulation of extrachromosomal L1 DNA copies. The synthesis of episomal L1 DNA copies (discussed below) and their stockpiling might be part of a cell “memory” mechanism aimed to accelerate noncanonical L1 DNA replication and dedifferentiation in response to a repetitive severe injury. It has been reported that repeated amputation of the axolotl limb results in accelerated regeneration [156], although the underlying mechanism is not understood.

Together, the analysis of L1 expression in a cell type-specific context shows that a correlation between a noticeable production of FL-L1 RNAs and cell phenotypic properties is not straightforward. Importantly, the production of FL-L1s might not necessarily always lead to the synthesis of both L1-encoded proteins and the formation of full-size RNPs. This may occur at the onset of meiosis and during the terminal stages of gametogenesis. The synthesis of full-size L1 RNPs in mitotically dividing cells appears to be strongly implicated in establishing gene expression profiles characteristic for totipotent/pluripotent and poorly differentiated cells.

### A shift in the current L1 paradigm: has the time come?

Barbara McClintock’s theoretical postulates on transposable genetic elements [22] were met with enduring reluctance, but this reluctance eventually evolved into acknowledgement of her discovery and revolutionary concept. Paradoxically, this now widely accepted concept seems to have become a barrier that impedes conceptual advances in L1 research.

The current L1 paradigm can be described as retrotransposition-centered: (i) retrotransposition is the only RT-dependent function of L1s considered so far; (ii) the drastic upregulation of L1s in early embryos and cancers is often deemed a non-specific response to general demethylation of the genome because it cannot be intended for retrotransposition, and other possible functions are usually not considered; (iii) the upregulation of endogenous L1s is usually thought to be a sufficient condition for retrotransposition despite the lack of a correlation between the abundantly expressed L1s and retrotransposition in the male germ line [19]; (iv) while retrotranspositionally active L1s are under scrutiny, retrotranspositionally inactive FL-L1s are neglected as elements that might be reverse transcribed and play an essential role in a cell; and (v) the attributed function of premature polyadenylation and splicing of L1 transcripts known to occur in many tissues is to defend against retrotransposition [140-142]; however, it is unlikely that L1 RNA is synthesized and processed merely to be non-functional.

The adherence to this retrotransposition-centered paradigm is reflected in the scarcity of research exploring other potential L1 RT-driven mechanisms. The adherence to the current paradigm is also evident in the interpretation of data demonstrating significant increases in L1 DNA copy number in the mouse zygote and cleaving embryos [27] as well as in regenerated axolotl limbs [155] as a result of numerous retrotransposition events. Although the reported increase in L1 DNA copy number may be partially caused by retrotransposition events, it is unlikely that retrotransposition is the sole L1 RT-dependent process in these cell systems. The activation of L1s in colorectal tumors is accompanied by 0 to17 new insertions per tumor sample [21]. Even if the degree of L1 activation in the early mouse embryo and regenerated axolotl limb is higher than in tumors, L1 retrotransposition rates in these cell systems are unlikely to be many times higher than in cancers. Consequently, other possible L1 RT-driven mechanisms are worth exploring.

One such mechanism could be the synthesis of extrachromosomal L1 DNA or L1 DNA-containing sequences. Abundant extrachromosomal circular L1 DNA-containing products have been found in yeast [157] and certain types of cancer cell lines [158]; however, the biological significance of these products remains unknown. The extrachromosomal L1 DNA copies might be the cause of significantly increased L1 DNA copy numbers in the regenerated axolotl limbs. The extrachromosomal L1 DNA copies may also be temporarily synthesized during early embryogenesis, thereby causing the amplification of L1 DNA copy number.

The second potential L1 RT-driven mechanism is the noncanonical L1 DNA replication proposed in this review. In early embryos, this mechanism could account for the qPCR-detectable amplification of L1 DNA copy number in time windows when only L1 DNA is replicated (by the noncanonical mechanism) in all or some embryo cells.

These two mechanisms may co-exist, interplay with each other, and be important for the establishment of an undifferentiated state of a cell. The noncanonical L1 DNA replication mechanism could serve as an important epigenetic mark that determines early replication of L1-rich developmentally regulated EtoL domains, whereas the formation of extrachromosomal L1 DNA copies could be an auxiliary molecular tool in support of it.

The proposed model implies that the noncanonical L1 DNA replication mechanism is normally executed in the totipotent and pluripotent cells of early embryos. Its initiation and primary specificity of the involved genomic domains is thought to be determined by a subset of L1 RNAs carried over into the zygote. The upregulation of the expression of FL-L1s at the two-cell stage and the gradual changes of L1 expression profiles during preimplantation development are deemed essential for the establishment of stage-specific gene-expression profiles. Noncanonical replication can potentially be triggered in differentiated somatic cells causing cell dedifferentiation and transformation, but not pluripotency because, the embryo- and cancer-specific profiles of FL-L1 RNAs are established under the influence of different factors.

From the standpoint of the proposed model, the unsolved L1-related issues mentioned in the second section of this review can be explained. Specifically, the co-expression of FL-L1 RNA and RT as well as DNA synthesis by reverse transcription, coinciding with the two-fold increase of the L1 DNA copy number in the early embryos, can be biologically explained. The model also explains the different responses of early-cleaving embryos and transformed cells to L1 knockdown and RT inhibition, specifically the complete cessation of divisions versus the continued proliferation at a lower rate. In the zygote, and to some extent the cleaving embryo, the specificity of a set of domains affected by noncanonical L1 DNA replication likely depends on L1 RNA delivered by the gametes. The degradation of L1 RNA by the L1-specific RNAi at the onset of embryogenesis does not allow the proper reprogramming of the genome. The same situation applies to the effect of RT inhibitors at this embryonic stage. The inability of a cell to proceed with proper spatial genome repositioning rather than the failure to complete a DNA replication round can be a consequence of L1 targeting. Those L1 RNPs that are bound to the genome for DNA replication may be less likely targets than cytoplasmic molecules. This notion is supported by the fact that the targeting of either L1 RNA or RT in transformed cells does not arrest the cells at a distinct point of the cell cycle. In poorly differentiated cancer cells synthesizing L1 RNPs, the experimental impediments to the putative noncanonical replication of L1s might switch the involved domains into a silent state. As a consequence, the gene expression profiles of transformed cells may change to those reminiscent of their normal counterparts. This may or may not cause a steady transition to normal cell functioning, depending on the “strength” of counteracting transforming factors and what point of the noncanonical replication mechanism has been targeted. Both of these aspects could explain the reinstated transformed phenotypes in a number of RT inhibitor-treated cancer cell lines after the withdrawal of the inhibitor. A clue as to why dedifferentiated transformed cells reprogram to their normal counterparts but not to other cell types upon the downregulation of L1s comes from the finding of lineage-dependent EtoL domains that are silenced during the specification of lineages [46]. These domains can more easily be reprogrammed back than pluripotency “indicator” EtoL domains. The changes of the replication timing of a portion of lineage-dependent EtoL domains might also be driven by the switch from noncanonical to canonical replication of the resident L1s. The majority of pluripotency “indicator” domains are likely to remain silent in most cancers, except for embryonal carcinomas and teratocarcinomas, whereas lineage-dependent EtoL domains might be commonly implicated in malignant dedifferentiation. Therefore, their silencing could favor the reprogramming of transformed cells into the pathway of their original lineage-specific differentiation. The proposed model can also explain why the epigenetic barrier established on the L1-rich EtoL pluripotency “indicator” domains is very stable. If L1 transcripts carried over by gametes into the zygote and synthesized in the early embryo under their direct influence do establish early replication of EtoL pluripotency “indicator” domains, the lack of such transcripts can impose a very stable silencing on these domains.

Some additional findings may or may not contradict the proposed model. First, it is not clear whether the results of cloning experiments fit the model. The model implies that the carried-over FL-L1 transcripts delivered by gametes and ORF2p are indispensable to set up the initial 3D genome architecture and replication timing program through noncanonical L1 DNA replication. Because the metaphase II oocyte, the common recipient used for somatic cell nuclear transfer [159], contains nuclear factors in its cytoplasm, FL-L1 transcripts might be available in the ooplasm if not bound to chromatin. ORF2p is present in the epididymal spermatozoa [27]. However, it is not clear whether ORF2p is lacking in the oocyte or a small amount of ORF2p is dispersed within the cytoplasm and is therefore barely detectable. The ability of the ooplasm to support reprogramming of transplanted nuclei of somatic cells to the totipotent state challenges the significance of L1 RT delivered by spermatozoa for genome reprogramming at the onset of embryogenesis.

Second, it is unclear whether the density of FL-L1s within the pluripotency “indicator” and certain lineage-dependent EtoL domains is high enough to control tethering and untethering of these domains with regard to the nuclear lamina. The average length of lineage-specific L1s peaks at regions with a GC content of 39–40% in the human and mouse genomes [3] suggesting that FL-L1s might accumulate in the pluripotency “indicator” domains, which have exactly the same GC content [45,51]. In contrast to the mouse genome, which has ~3000 potentially active FL-L1s [160], the human genome harbors only ~85 retrotranspositionally active copies of ~7000 FL-L1s [134]. Nevertheless, some retrotranspositionally inactive FL-L1s might be capable of reverse transcription *in vivo*. Because the number of FL-L1s capable of reverse transcription remains unclear, it is perplexing whether the subset of reverse-transcribed FL-L1s is large enough to establish transcriptional competence for a large cohort of genes.

The hypothesis proposed in this review is testable. The simplest experimental model to test whether noncanonical L1 DNA replication occurs would be one-cell mouse embryos. Two factors favor this experimental model: the RT-dependent phase of DNA synthesis in zygotic pronuclei precedes the DNA polymerase-dependent DNA replication, and the time frames of these events have been defined [27]. Two sequential labelings of synthesizing DNA with halogenated nucleotides (e.g., IdU and CldU) during these two phases, and the subsequent visualization of their incorporation by fluorescently labeled antibodies on stretched DNA fibers combined with parallel L1 DNA-specific fluorescence *in situ* hybridization (FISH), is expected to be informative. The labeling protocol introduced for the single-molecule analysis of replicated DNA [161,162] can be coupled with proper modification of the method of microfluidic extraction and the stretching of DNA from single nuclei [163]. A modification of the method of microfluidic stretching of DNA is required to provide better resolved DNA fibers. The lack of data regarding whether the RT-dependent phase of DNA synthesis exists in ESCs and certain transformed cell lines, whether it overlaps with or precedes the DNA polymerase-dependent phase, and whether the cells would be able to resume DNA synthesis after the withdrawal of aphidicolin makes the suitability of the same approach suggested for one-cell embryos uncertain. Additionally, ChIp-seq of either BrdU-labeled nascent DNAs or nascent DNA-ORF2p complexes obtained from aphidicolin-treated ESCs and transformed cell lines could be considered. The knockdown of specific subsets of FL-L1s, and the inhibition of L1 RT in ESCs and transformed cell lines, followed by analyses of replication timing, gene expression, and S/MAR profiles at the genomic scale could clarify whether activated FL-L1s regulate gene expression through the establishment of replication timing and S/MAR profiles.

Prompted by anti-tumor effects of RT inhibitors in experimental models, an attempt was made to employ nevirapine for the treatment of non-HIV cancer patients in a small clinical trial [164]. This clinical trial was also based on positive outcomes of RT inhibitor-based treatment regimes for HIV-related tumors, which could partially be attributed to a direct anti-cancer activity of the drugs [165]. However, this approach did not lead to the anticipated result because nevirapine appeared to be toxic to some non-HIV-infected cancer patients [164] and was perhaps a suboptimal inhibitor of L1 RT. From the standpoint of the model proposed here, targeting the L1 RNP-driven process at the RT level might be an ineffective means to obtain the irreversible differentiation of cancer cells even if highly specific anti-L1 RT drugs are used. Preventing the licensing of sites of noncanonical replication might be a more fruitful approach to obtain sustained differentiation of cancer cells. Uncovering the biological significance and the mechanism of L1 RT-dependent DNA synthesis would inform the development of highly targeted anti-cancer therapies and new approaches to control the reprogramming of differentiated cells into iPSCs. In addition, more detail on the sequences of the FL-L1 RNAs forming the full-size L1 RNPs in cancers would open a new avenue in the field of cancer biomarkers.

## Conclusions

Available data demonstrate that several L1-related phenomena cannot be explained within the framework of the current retrotransposition-centered L1 paradigm. A novel concept is required to explain the nature of massive L1-linked reverse transcription at the onset of embryogenesis and how abundantly expressed FL-L1 RNA and RT can globally control the epigenetic state of a cell. A revised L1 paradigm should put into focus the possibility of L1 RT-driven biologically significant processes other than retrotransposition.

A new concept of noncanonical L1 DNA replication that could exist in early embryos, ESCs, and certain types of cancer has been introduced in this article. This proposed model links undifferentiated states of a cell, such as totipotency, pluripotency, and regeneration-/cancer-related dedifferentiation to this mechanism. The hitherto unexplained phenomena that demonstrate crucial though different outcomes of the downregulation of L1s and RT in early embryos and cancers can also be explained. First, the proposed model assigns a biological function to upregulated FL-L1s, L1-encoded proteins, and L1-linked reverse transcription. Second, it suggests how the L1 RNP-driven process could potentially result in transcriptional competence of specific domains of the genome that harbor genes associated with undifferentiated states. Moreover, the model demonstrates how the L1 RNP-driven process could integrate with other fundamental processes in the nucleus. Finally, the model shows how the whole system might be regulated in development and dysregulated in cancer.

An important aspect of this novel concept is that it links retrotransposition of endogenously expressed L1s to the putative noncanonical L1 DNA replication. Evidence supporting this claim is provided. Endogenous L1 retrotransposition is clearly non-random, but seems biased to a similar sequence environment. In addition, L1 retrotransposition mainly occurs in proliferating undifferentiated embryonic and cancer cells, but not in all types of cells where L1s and FL-L1s are abundantly expressed.

Although the current model of DNA replication seems robust, it should be retested in specific genome locations (distinct FL-L1 sequences) in early embryonic and cancer cell systems. This is suggested by the failure of the prevailing L1 paradigm to explain several important L1-related phenomena and the plausibility of the proposed model of noncanonical L1 DNA replication.

## Reviewers’ reports

### Reviewer 1: Dr. Philip Zegerman, Wellcome Trust/Cancer Research UK Gurdon Institute, University of Cambridge, Cambridge, UK (nominated by Dr. Orly Alter, University of Utah, Salt Lake City, USA)

Understanding the physiological roles of transposable elements is an important biological question. This review aims to link the transcription and duplication of L1 elements to other cellular processes including replication timing and changes in the chromatin state.

This review would have benefitted from a clearer and more precise analysis of key experiments in a defined manner. Instead sweeping conclusions are made from some sparse data e.g. “the data available at this time show no evidence that the massive nuclear reverse transcription occurring in early embryos is DNA replication independent.” p.24, yet the aphidicolin experiment in ref 27 clearly demonstrates the opposite.

Response: *Indeed, data used in this review are often insufficient for definite conclusions. This is not surprising because the issues discussed and questions raised in this paper have never been addressed experimentally. However, when sparse data accumulate to the necessary threshold, I think it is timely to draw the attention of the research community to interpretational or conceptual issues. Some findings have been reported by authors as minor details, but they have a certain value when viewed in a new context or linked with other data and, therefore, are worth being included in this review.*

The requirement for well-supported conclusions to be based on strong evidence is appropriate for a paper that employs the deductive approach. This review, on the contrary, is an inductive paper. I recognize the original text contained some generalizations that could sound as sweeping conclusions, and thus I have critically reassessed the text and changed wording in some instances.

I do not agree with the latter comment regarding the text on p. 24. The concluding sentence of the section that is cited is taken out of the context. It summarized the there main points of the preceding discussion: 1) the interpretation of aphidicolin-resistant abacavir-sensitive synthesis of DNA by reverse transcription in the zygote as DNA replication independent was based on the current concept of DNA replication, which may not be comprehensive; 2) there were some overlooked timing issues related to initiation of DNA synthesis in the zygote, which question the conclusion made in ref. 27; and 3) there were some drawbacks in the design of the experiments described in ref. 27, which made the experiments inconclusive in terms of whether the DNA synthesis by reverse transcription in the zygote was DNA replication dependent or independent.

I would like to emphasize that the endurance of a particular scientific hypothesis does not make it an ultimate truth. It is reasonable to interpret new results on the basis of a particular hypothesis until some data that support new testable predictions are obtained. I suggest that this is the case with the current concept of DNA replication. To this end, I have strengthened this point in the paper. I have also made small changes to clarify the point that experiments in ref. 27 were inconclusive with respect to their claim that the DNA synthesis by reverse transcription in the zygote was DNA replication independent.

Another example would be the statement “these data suggest that ORCs are highly likely to bind to G4 structures of L1s”. p.28.

Response: *I have clarified this statement by including an additional point from the preceding discussion: “these data suggest that ORCs are highly likely to bind to G4 structures of those L1s that tether to the nuclear matrix.” The logic underlying this statement is below. About 225,000 active origins (90% of all active origins) are associated with G4s*[80]*; however, the number of inactive G4-bound ORCs is not known. L1s are a substantial source of G4-forming sequences. Given that the human genome contains ~516,000 L1s, most of which are truncated at the 5′ (not at the 3′) end*[2]*, and all L1s with intact 3′ UTRs contain a G-forming tract*[95]*, the number of G4-forming sequences can be significantly greater than current estimates of 375,000 [Todd AK et al., Nucleic Acids Res, 2005, 33:2901–2907]. Despite the growing interest in the G4-ORC link, no one has attempted to estimate what portion of G4s associated with ORCs is represented by L1-derived G4s. With so many unknowns, a landmark for future investigations could be what we can see in the nucleus. The abundance of L1s among MARs and preferential colocalization of origins and MARs suggest that chromatin is organized in such a way that many L1s likely serve as MARs and ORC binding sites at the same time.*

I would urge the author to reassess the review and re-balance the description and interpretation of the experimentation. This should be married with a considerable reduction (50%) in the length of the review to allow it to be accessible to as wide a community of scientists as possible.

Response: *I appreciate the concerns with respect to making the manuscript more accessible to as wide a community as possible; however, I believe the paper will lose its value to specialists as well as the integral view if the experimental or interpretative components are so drastically pruned.*

This is a multidisciplinary work that integrates experimental data from a number of fields. Therefore, some introductory information regarding L1 biology, replication timing, etc., are worth inclusion so the paper is accessible to a multidisciplinary readership. Moreover, retaining the experimental data that might be considered as non-key facts is important. From the perspective of the introduced concepts, the whole picture that emerges from the integration of the key and non-key facts is a more convincing piece of information than several findings standing alone. This is important because the concepts and interpretation of certain experimental data are provocative.

This review is not in a narrative style. As mentioned above, this is an inductive paper that purports a considerable interpretative component. The interpretative portion of the manuscript is as important as the experimental with respect to integrating the experimental material, introducing alternative explanations, pointing out issues pertinent to the current L1 paradigm, proposing conceptual changes, and examining how the available data fit the model. The discussion of potential links between the phenomena that have never been thought linked opens new avenues for research. I believe there is some value in this intellectual contribution.

In recognition of the length issue, I have deleted a few details such as the names of genes that changed their expression levels in response to the downregulation of L1s in A-375 melanoma cells and the concentrations of the RT inhibitors used to reprogram cancer cell lines and to assess their effects on retrotransposition of L1s. Some redrafting has also been done to make some paragraphs more concise.

Quality of written English: Acceptable

### Reviewer 2: Dr. I King Jordan, Georgia Institute of Technology, Atlanta, USA

The manuscript on the functional significance of (potentially) non-canonical L1 expression and replication by Ekaterina Belan is a provocative mix of a review article and a hypothesis paper. The author extensively reviews current experimental evidence on the role of L1 reverse transcription in early embryos and cancer in light of recent findings on genome regulation, organization and epigenetics. A key to understanding the authors approach is the desire to explore novel functional roles for L1s that do not fit within the current paradigm of L1 biology, which focuses mainly on retrotransposition dynamics and host genome mechanisms for the repression of transposition.

The search for a functional role of L1s rests on the author’s notion that since the main role of L1s is not the introduction of genomic variation “it is logical to assume that an important function (or functions) of L1s remains to be discovered.” While this kind of teleological thinking is tempting, one does not need to invoke a direct function of L1s to explain their existence and abundance in the genome (or their regulatory anomalies for that matter). As is held by the selfish DNA theory, the existence of such elements can be explained solely by their ability to out-replicate the genomes in which they reside.

Response: *This is a very good point. I have revised the paragraph to include consideration of the evolutionary aspect.*

Having said that, once having established themselves in their hosts’ genomes, it is almost certainly the case that elements of this kind can have a profound effect on genome function. Accordingly, what the author refers to as the current ‘retrotransposition-centered paradigm’ of L1 biology may indeed lead to interpretations of experimental evidence that are markedly different from those offered in this manuscript. As such, the alternative hypotheses and views proposed here do seem to cover new ground, are thought provoking, lead to testable predictions (to some extent), and are thus worthy of publication in Biology Direct.

Some of the interpretations of L1 experimental data presented here are likely to be controversial, particularly to the extent that they differ from interpretations offered by the authors of the studies that generated the data. Thus, the paper has the potential to generate a substantive response and a potentially interesting discussion in the field and/or the literature. To her credit, the author does provide specific experimental tests of her models as they relate to the occurrence of non-canonical L1 DNA replication and the role of full-length L1 expression in genome regulation.

Finally, it is worth noting that the topics covered in this review, and in particular the experimental tests proposed, could have biomedical relevance with respect to the link between L1 reverse transcriptase-dependent DNA synthesis and cancer and/or stem cells. A better understanding of this phenomenon could hold promise for the development of L1 related anti-cancer therapies and/or novel methods for the reprogramming of differentiated cells to pluripotent stem cells.

Quality of written English: Acceptable

### Reviewer 3: Dr. Panayiotis (Takis) Benos, University of Pittsburgh, Pittsburgh, USA

This reviewer provided no comments for publication.

## Abbreviations

LINE: Long interspersed nuclear element; LINE-1(L1): Long interspersed nuclear element-1; FL-L1: Full-length L1; RT: Reverse transcriptase; EN: Endonuclease; RNP: Ribonucleoprotein; cDNA: Complementary DNA; L1Hs: Subfamily of human-specific (from *Homo sapiens*) L1 elements (also known as L1PA1); L1PA2 L1PA3, L1PA4, L1PA6, L1PA7: Subfamilies of primate-specific L1 elements; Ta-1: Transpositionally active subfamily of human L1 elements (also known as L1Hs- Ta1); UTR: Untranslated region; ORF: Open reading frame; ORF1: Open reading frame 1; ORF2: Open reading frame 2; ORF1p: Open reading frame 1 protein; ORF2p: Open reading frame 2 protein; bp: Base pair(s); kb: Kilobase pairs; Mb: Megabase pairs; siRNA: Small interfering RNA; RNAi: RNA interference; S/MAR: Scaffold/matrix attachment region; SAR: Scaffold attachment region; MAR: Matrix attachment region; ERV: Endogenous retrovirus; HERV-K: Human endogenous retrovirus family; MuERV-L: Murine endogenous retrovirus-like element; AML: Acute myeloid leukemia; ICM: Inner cell mass; ESC: Embryonic stem cell; hESC: Human embryonic stem cell; mESC: Mouse embryonic stem cell; iPSC: Induced pluripotent stem cell; EpiSC: Stem cell derived from the epiblast; NPC: Neural precursor cell; EtoL: Replication timing change from early to late S; LtoE: Replication timing change from late to early S; Xa: Active X chromosome; Xi: Inactive X chromosome; BrdU: 5-Bromodeoxyuridine; IdU: Iododeoxyuridine; CldU: Chlorodeoxyuridine; HCG: Human chorionic gonadotropin; qPCR: Quantitative real-time polymerase chain reaction; INM: Inner nuclear membrane; ORC: Origin recognition complex; ORCA: ORC-associated protein; HP1: Heterochromatin protein 1; G4: G-quadruplex; H3K9me3: Histone H3 trimethylated at lysine 9; H3K9ac: Histone H3 acetylated at lysine 9; TDP: Timing decision point; 3D: Three-dimensional; wt: Wild type; R loop: RNA•DNA displacement loop; ssDNA: Single-stranded DNA; dsDNA: Double-stranded DNA; FISH: Fluorescence *in situ* hybridization; ChIp-seq: Chromatin immunoprecipitation followed by high-throughput DNA sequencing; HIV: Human immunodeficiency virus.

## Competing interests

The author declares that she has no competing interests.

## Supplementary Material

Additional file 1 Extended Discussion. Click here for file
