# Supplementary material for: LINEs of evidence: noncanonical DNA replication as an epigenetic determinant
Source: Biol Direct. 2013 Sep 13;8:22. doi: 10.1186/1745-6150-8-22 (PMC3868326; doi:10.1186/1745-6150-8-22)
Supplement: Additional file 1 — Extended Discussion. [file 1745-6150-8-22-S1.pdf]

Additional file 1.

LINEs of evidence: noncanonical DNA replication as an epigenetic determinant

Ekaterina Belan

Extended Discussion.

Five methodological issues are considered. (i) Synthesis of FL-L1 RNAs, which is common in many normal tissues and transformed cell lines, is usually followed by various processing of these RNAs to shorter forms [140-142]; therefore, Northern blot data based on one or two L1-specific probes might be inconclusive. (ii) ORF2p and ORF1p can potentially be synthesized on spliced and prematurely polyadenylated L1 transcripts *in vivo* [140,142] and, consequently, the detection of ORF1p and ORF2p by specific antibodies cannot be interpreted as evidence of the presence of FL-L1 transcripts and full-size L1 RNPs if the data are not supported by parallel studies of the L1 RNAs. (iii) The results of L1 RNA studies suggest that truncated forms of ORF1p and ORF2p can be expressed [140,142,143]. Indeed, a truncated form of ORF2p has been found *in vivo* by antibodies generated to detect the full-size form [143]; therefore, positive immunostaining for ORF1p and ORF2p should not be interpreted as the detection of full-size L1 proteins if the results are not supported by protein analysis. (iv) Several forms of ORF1p of different molecular weights and, potentially, with different functional roles, are known to be synthesized in a cell type-specific and developmentally regulated manner [16-18]. These forms are thought to result from both ORF1 sequence variation in the L1 multigene family and post-translational modifications [18], a fact that raises uncertainty as to whether some forms of ORF1p can remain undetectable by the commonly used ORF1p-specific antibodies. (v) The difficulty in detecting ORF2p *in situ* using

antibodies, as encountered in some studies, is thought to be due to low antibody specificity [1,143]. However, the possibility of premature polyadenylation of the L1 RNA, resulting in the absence of detectable ORF2p, raises concerns about the correct interpretation of negative results.
